# In-vivo histology of Parkinson’s disease using quantitative multiparametric mapping

**DOI:** 10.1038/s41531-026-01329-4

**Published:** 2026-03-31

**Authors:** Marta M. Pokotylo, Martin Göttlich, Laura Schmidt, Helena Sophia Weingarten, Lydia Dirksen, Lea Albrecht, Norman Griebner, Jan Uter, Julia Henkel, Norbert Brüggemann, Jannik Prasuhn

**Affiliations:** 1 Section of Movement Disorders, Department of Neurology, University of Medical Center Schleswig-Holstein, Campus Lübeck, Lübeck, Germany; 2 Center for Brain, Behaviour, and Metabolism, University of Lübeck, Lübeck, Germany; 3 Institute of Medical Psychology, University of Lübeck, Lübeck, Germany; 4 Institute of Neurogenetics, University of Lübeck, Lübeck, Germany; 5 Department of Neurology, Johns Hopkins University School of Medicine, Baltimore, MD USA; 6 F.M. Kirby Research Center for Functional Brain Imaging, Kennedy Krieger Institute, Baltimore, MD USA

**Keywords:** Diseases, Neurology, Neuroscience

## Abstract

Parkinson’s disease (PD) is a progressive neurodegenerative disease caused by the loss of dopaminergic neurons; however, growing evidence indicates the widespread involvement of cortical regions underlying motor and non-motor symptoms. In this study, we evaluated multiparametric mapping (MPM) as a non-invasive imaging technique for the detection of microstructural brain alterations in PD. We assessed 31 patients with idiopathic PD (PwPD) and 68 healthy controls (HCs) utilizing MPM-derived longitudinal relaxation rate (R1), effective transverse relaxation rate (R2*), proton density, and magnetic transfer saturation (MTsat). We performed a whole-brain voxel-based quantification (VBQ) and multiple linear regression analysis to assess group differences and associations with the clinical phenotype. Lower MTsat within the left superior frontal gyrus (SFG) predicted motor symptom severity in PwPD, while higher R2* values in the right SFG were associated with higher levodopa-equivalent daily dose. Higher R2* values in the posterior cingulate gyrus and lower proton density in the left superior parietal lobule were associated with a stronger cognitive impairment in PwPD. Additionally, numerous clusters presented with group differences across multiple MPM modalities, including the supplementary motor area and cingulate cortex. Overall, we successfully replicated previously documented cortical microstructural changes in PwPD, presenting MPM as a sensitive tool for the detection of disease-specific alterations. This study further underscores the utility of MPM for monitoring disease progression through biologically informed measures, opening avenues for further research.

## Introduction

Neurological disorders are among the leading causes of disability worldwide, with Parkinson’s disease (PD) being the second most prevalent and fastest-growing neurodegenerative disease^1^. PD primarily impacts motor functions but is increasingly recognized to also affect cognitive and affective domains, all of which significantly diminish the quality of life for patients with Parkinson’s disease (PwPD)^2^. As current gold-standard symptomatic treatments fail to stop neuronal loss, the need for improved tools that deepen our understanding of PD pathology and support personalized therapeutic strategies is becoming more urgent. The onset of PD symptomatology is preceded by early changes, including alpha-synuclein aggregation, mitochondrial dysfunction, neuroinflammation, and iron accumulation^3^. While the substantia nigra (SN) is recognized to be the primary site of pathology, increasing evidence demonstrates that these pathogenic processes result in widespread microstructural changes, including cortical thinning and white matter (WM) tract alterations^4^.

In addition to the clinical examination, neuroimaging is routinely employed for the differential diagnosis and for establishing markers of disease progression in PwPD^5^. An evident example is the use of nuclear imaging techniques such as positron emission tomography (PET) and single-photon emission computed tomography (SPECT) to assess pre- and post-dopaminergic neurotransmission in the brain, confirming loss or dysfunction of dopaminergic neurons, aiding in confirming SN degeneration, and differentiating Parkinsonian syndromes^6^. However, these approaches are invasive, limited to only functional insights, falling short in probing biologically interpretable microstructural changes underlying observed functional alterations in PwPD. Magnetic resonance imaging (MRI) offers a valuable, non-invasive approach to assess both morphological and functional alterations in PwPD. However, conventional MRI has limited sensitivity and is only 50% sensitive in differentiating PD from Parkinsonian syndromes, limiting its use for the detection of subtle microstructural changes that precede significant neuronal loss and clinical presentation of PD^7^. In this context, quantitative MRI (qMRI) techniques, such as multiparametric mapping (MPM), present a potentially superior imaging alternative with higher sensitivity, providing qualitative estimates of biophysical tissue properties, enabling enhanced interpretation of brain microstructure and composition at the cellular level^8^.

MPM enables voxel-wise estimation and quantification of tissue-specific biophysical properties, such as longitudinal relaxation rate (R1), effective transverse relaxation rate (R2*), proton density, and magnetic transfer saturation (MTsat), by modeling signals from multi-contrast gradient-echo acquisitions, simultaneously correcting for hardware-related confounding factors^9–12^. These quantitative maps facilitate the in vivo investigation of tissue characteristics, as R1 and MTsat are sensitive to myelin content, proton density to water content, and R2^*^ reflects iron accumulation, increasing the specificity of PwPD data interpretation to levels previously only achievable through histology, labeling MPM as an in vivo histology tool^13^. Since R2* is sensitive to iron deposition, an established factor in PD pathogenesis, MPM becomes an even more valuable tool, integrating R2* with complementary measures (R1, proton density, and MTsat), providing deeper insights into PD-specific pathological processes^14^. Although detailed microscopic analysis in PwPD is usually performed with ex vivo histology, such procedures reveal changes after most degenerative processes have already occurred, limiting their clinical relevance. Contrarily, MPM offers in vivo access to earlier pathogenic processes, providing a superior approach^8^.

Despite its potential, MPM imaging remains largely overlooked in clinical research, and its use in neurodegenerative diseases such as PD remains limited due to the technical complexity of implementing MPM. The recently developed open-source hMRI toolbox allows for the standardized reconstruction and processing of quantitative maps, increasing MPM clinical accessibility^8^. The standardized procedure of the toolbox allows for the derivation of MPM parameters while minimizing partial-volume effects, increasing the data comparability.

As MPM-derived biological insights hold a significant potential to advance our understanding of PD pathology, we aimed to utilize fully quantitative MPM maps to investigate gray matter (GM) and WM microstructural alterations in PwPD^12,15,16^. In this cross-sectional study, we examined MPM’s utility in detecting regional cortical microstructural differences in PwPD compared to healthy controls (HCs). Given the extensive evidence demonstrating whole-brain cortical neuronal loss, demyelination, and structural reorganization, in addition to SN pathology, we performed an exploratory analysis to assess whether MPM-derived data would reveal regional microstructural differences associated with the clinical presentation of PwPD^17–19^. This supports the application of MPM in clinical settings as a promising tool that could facilitate the development of personalized therapeutic approaches based on individual alterations and support in PwPD stratification.

## Results

### Demographic and clinical assessment

After the quality control procedure, we included 99 participants, consisting of 31 PwPD (mean age: 65.5 ± 8.1; 18 females) and 68 HCs (mean age: 65.0 ± 9.3; 40 females). The mean age did not differ between groups (independent two-tailed *t*-test, *p* = 0.720). Both groups demonstrated a comparable sex distribution, with no significant difference (PwPD: females: 18/31; HCs: females: 40/68; Chi-squared test: χ^2^ = 0.017, df = 1, *p* = 1.000). The PwPD cohort had an intermediary disease duration (4.5 ± 4.3 years) and, on average, a moderate disease severity, informed by the Movement Disorders Society Unified Parkinson’s Disease Rating Scale, subscore III (MDS-UPDRS-III: 28.6 ± 15.5) and Hoehn and Yahr stage (median [range]: 2 [1,2]). The cognitive domain in PwPD was assessed by the Montreal Cognitive Assessment (MoCA), revealing an average 26.7 ± 2.3 MoCA score. The average levodopa-equivalent daily dose (LEDD) was 513 ± 366 (see Table 1).

**Table 1:** Demographic and clinical characteristics

	PwPD	HCs	*p*-value
Number	31	68	
Male/Female	13/18	28/40	1.000^a^
Age (years)	64.9 ± 9.3 [60.00–72.00]	65.6 ± 8.1 [56.00–71.00]	0.720^b^
MDS-UPDRS-III	28.6 ± 15.5 [19.00–34.00]	n.a.	
LEDD (mg/d)	512.6 ± 365.6 [300.00–842.50]	n.a.	
Disease duration (years)	4.5 ± 4.3 [1.50–7.00]	n.a.	
Hoehn and Yahr stage	2 [1,2	n.a.	
MoCA	26.7 ± 2.3 [25.50–28.00]	n.a	

Mean values and standard deviations are quoted. Values in square brackets represent the interquartile range.

*HCs* healthy controls, *LEDD* levodopa equivalent daily dose, *MoCA* Montreal Cognitive Assessment, *MDS-UPDRS-III* Movement Disorders Society’s Unified Parkinson’s Disease Rating Scale (subscore III).

^a^according to a χ^2^-test.

^b^two-sample *t*-test applied.

Once we ensured that both cohorts were well-matched, we proceeded with whole-brain voxel-based quantification (VBQ) analysis to identify brain clusters with significant differences in each MPM modality between PwPD and HCs, separately for GM and WM (see Supplementary Fig. 1). We tested two contrasts: higher values in PwPD (PwPD > HCs) and higher values in HCs (HCs > PwPD). To link group-level microstructural changes with the clinical presentation of PwPD, we performed correlation and multiple linear regression analyses using mean parameter values from clusters demonstrating significant group differences that were anatomically and functionally relevant to the clinical measure of interest. Clinical presentation of PwPD was characterized by MDS-UPDRS-III, LEDD, disease duration, and MoCA scores. Additionally, we performed exploratory cross-modal correlation analyses to investigate whether the clusters with identified significant group differences in one modality correlate with values from other MPM-derived modalities, to probe the degree of overlap.

### Increased R1 in cingulate and motor regions in PwPD

**Fig. 1:**
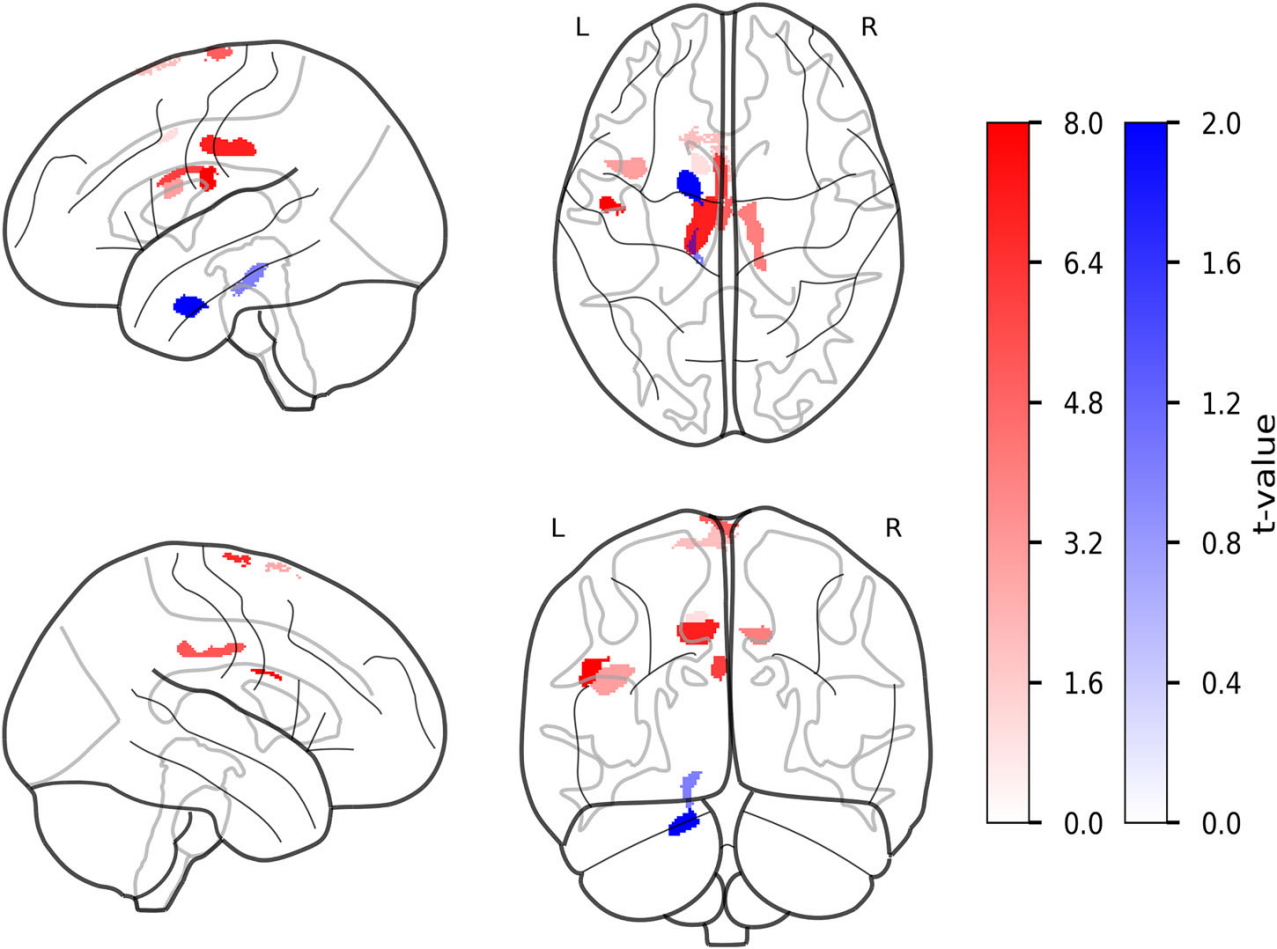
Group differences in R1 values between PwPD and HCs in gray matter. The statistical parametric maps from voxel-wise group comparison of R1 values are presented, displayed on a standard brain template in sagittal, axial, and coronal views. The statistical maps show t-statistics, where red clusters have higher R1 values in PwPD (PwPD > HCs), and blue clusters have higher R1 values in HCs (HCs > PwPD). The color bars reflect voxel-wise t-statistics, with higher absolute *t*-values indicating stronger group differences only. Cluster-defining threshold was set to *T* = 3.18 (*p* < 0.001, uncorrected) with a cluster extent threshold of *k* ≥ 20 voxels. FWE correction was applied at the cluster levels for PwPD > HCs contrast: FWEc = 140, df = [1,94] and HCs > PwPD contrast: FWEc = 152, df = [1,94]. df degrees of freedom, FWE family-wise error, HCs healthy controls, k number of voxels, L left hemisphere, PwPD patients with Parkinson’s disease, R right hemisphere, R1 longitudinal relaxation rate, T t-statistic.

We compared R1 values between PwPD and HCs at a cluster-defining threshold of *p* < 0.001 (uncorrected), with family-wise error (FWE) correction applied at the cluster level. We identified nine clusters with higher R1 values in the PwPD group (FWEc = 156), with left middle cingulate cortex (lMCgG) passing the extent of 1000 voxels (*k* = 1280, *T* = 3.60, p_FWE_ = 0.000). Only two clusters had higher R1 values in HCs (FWEc = 153), including left ventral diencephalon (lVD, *k* = 153, *T* = 4.87, p_FWE_ = 0.028) and left entorhinal area (*k* = 457, *T* = 4.20, p_FWE_ = 0.000, see Fig. 1 and Table 2). Multiple linear regression analysis revealed no association between identified clusters and MDS-UPDRS III, LEDD, as well as disease duration and MoCA scores.

**Table 2:** Clusters with significant differences in R1 values between PwPD and HCs

	Cluster level				Peak level		mm	mm	mm
	*P*_FWE-corr_	*q*_FRD-corr_	*K*_E_	*P*_uncorr_	*T*	(*Z*_E_)			
**PwPD** > **HCs**									
lSMA	0.000	0.000	466	0.000	5.80	5.56	0	–11	76
IOpIFG	0.005	0.002	696	0.000	4.77	4.51	–37	6	24
lMCgG	0.000	0.000	1280	0.000	4.54	4.31	–9	–13	37
lCO	0.001	0.000	258	0.000	4.24	4.05	–48	–9	23
lMTG	0.018	0.003	164	0.000	4.05	3.88	–42	–60	12
lSMA	0.005	0.001	199	0.000	3.95	3.79	–13	8	43
lTrIFG	0.025	0.004	156	0.000	3.87	3.72	–44	28	9
rMCgG	0.000	0.000	627	0.000	3.76	3.62	12	–20	36
lMCgG	0.000	0.000	279	0.000	3.60	3.48	–2	6	27
**HCs** > **PwPD**									
lVD	0.028	0.016	153	0.000	4.87	4.59	–14	–24	–17
lEnt	0.000	0.000	457	0.000	4.20	4.01	–13	3	–25

The table presents the clusters demonstrating significant group differences in R1 values at the cluster level.

*FDR-corr* false-discovery rate-corrected, *FWE-corr* family-wise error-corrected, *HCs* healthy controls, *K*_*E*_ cluster extent, *lCO* left central operculum, *lEnt* left entorhinal area, *lMCgG* left middle cingulate gyrus, *lMTG* left middle temporal gyrus, *lOpIFG* left opercular part of the inferior frontal gyrus, *lSMA* left supplementary motor area, *lTrIFG* left triangular part of the inferior frontal gyrus, *lVD* left ventral diencephalon, *PwPD* patients with Parkinson’s disease, *R1* longitudinal relaxation rates, *rMCgG* right middle cingulate gyrus, *T* t-statistic, *Uncorr* uncorrected, *Z*_*E*_ equivalent *z*-score.

### Altered R2* are associated with treatment status and cognitive state in PwPD

**Fig. 2:**
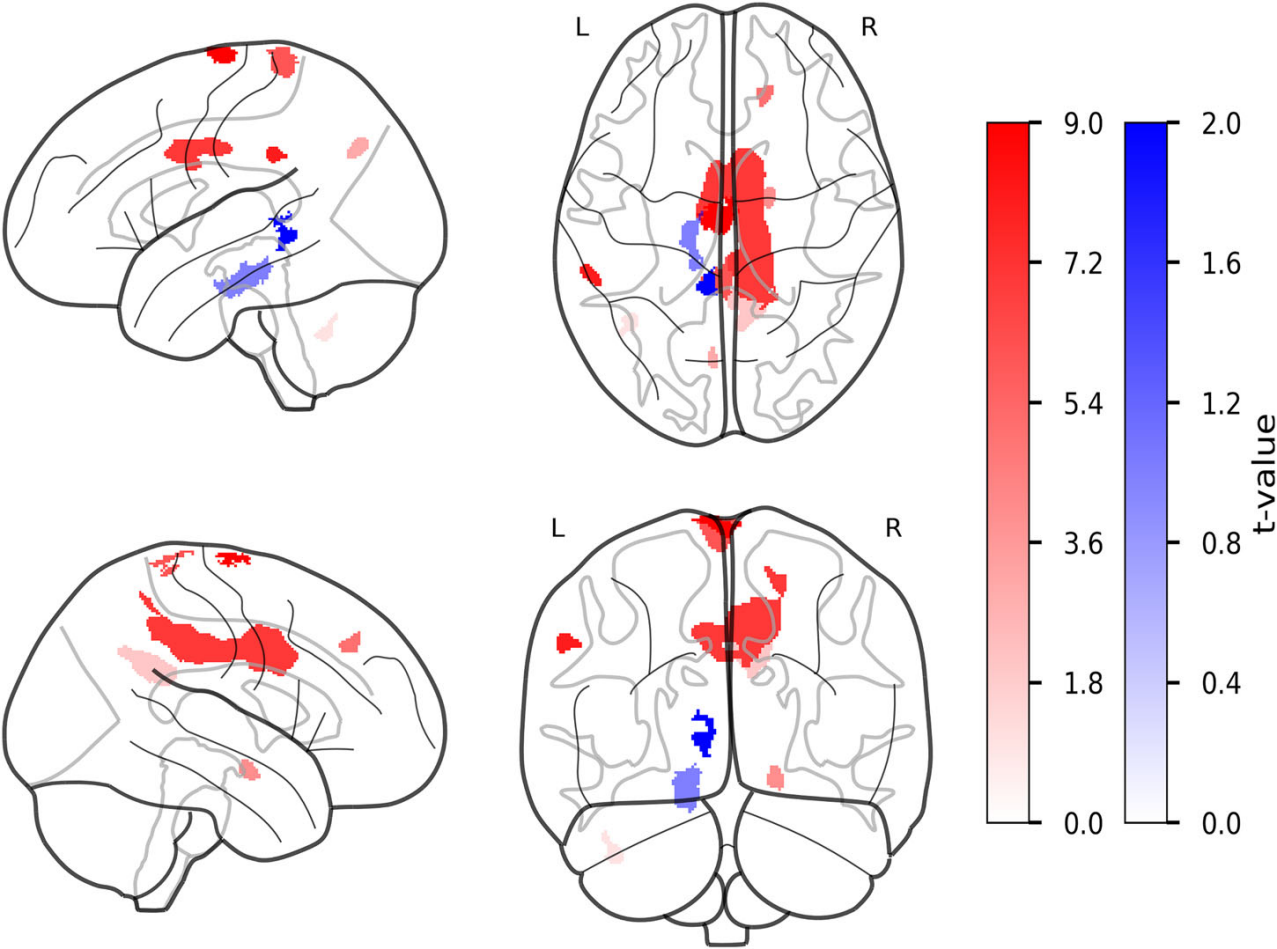
Group differences in R2* values between PwPD and HCs in gray matter. The statistical parametric maps from voxel-wise group comparison of R2* values are presented, displayed on a standard brain template in sagittal, axial, and coronal views. The statistical maps show t-statistics, where red clusters have higher R2* values in PwPD (PwPD > HCs), and blue clusters have higher R2* values in HCs (HCs > PwPD). The color bars reflect voxel-wise t-statistics, with higher absolute *t*-values indicating stronger group differences only. Cluster-defining threshold was set to *T* = 3.18 (*p* < 0.001, uncorrected) with a cluster extent threshold of *k* ≥ 20 voxels. FWE correction was applied at the cluster levels for PwPD > HCs contrast: FWEc = 154, df = [1,94] and HCs > PwPD contrast: FWEc = 253, df = [1,94]. df degrees of freedom, FWE family-wise error, HCs healthy controls, k number of voxels, L left hemisphere, PwPD patients with Parkinson’s disease, R right hemisphere, R2* effective transverse relaxation rate, T t-statistic.

For the group comparison of R2* values, cluster-level FWE correction resulted in an extended threshold of FWEc = 154 for PwPD > HCs contrast and FWEc = 253 for HCs > PwPD contrast. We identified eight clusters with higher R2* values in PwPD and six clusters with higher R2* values in HCs (see Fig. 2 and Table 3). For the assessment of the association between identified clusters’ R2* values and LEDD among the PwPD group, we used R2* values from the lVD, right MCgG (rMCgG), left posterior gyrus medial segment (lMPoG), left supramarginal gyrus, right superior frontal gyrus (rSFG), and left cerebellum exterior (lCE). Multiple regression analysis revealed that only lower R2* values in the rSFG were associated with greater LEDD intake among PwPD (*β* = 157.83, *p* = 0.037, R^2^ = 0.33, R^2^_adj_ = 0.13, see Table 4). In addition, we performed the same analysis using the lMPoG, right posterior cingulate gyrus (rPCgG), and left PCgG (lPCgG), left supramarginal gyrus, and right amygdala as predictors of cognitive state in PwPD, informed by MoCA scores. The overall model explained only a moderate proportion of variance (R^2^ = 0.38; R^2^_adj_ = 0.25), with lower R2* values within a cluster in the lMPoG associated with greater MoCA scores in PwPD (*β* = 0.6389, *p* = 0.034).

**Table 3:** Clusters with significant differences in R2* values between PwPD and HCs

	Cluster level				Peak level		mm	mm	mm
	*P*_FWE-corr_	*q*_FRD-corr_	*K*_E_	*P*_uncorr_	*T*	(*Z*_E_)			
**PwPD** > **HCs**									
lSMA	0.002	0.001	612	0.000	6.01	5.51	0	–11	76
rMCgG	0.000	0.000	8673	0.000	5.19	4.85	4	3	40
lMPoG	0.000	0.000	579	0.000	4.91	4.62	–3	–40	76
rPCgG	0.000	0.000	1135	0.000	4.27	4.07	13	–46	29
rAmygdala	0.002	0.001	232	0.000	4.26	4.06	17	–4	–14
lSMG	0.014	0.003	179	0.000	4.10	3.93	–58	–36	36
rSFG	0.043	0.008	149	0.000	3.92	3.77	14	38	40
lCE	0.028	0.006	161	0.000	3.54	3.42	–40	–57	–40
**PwPD** < **HCs**									
lVD	0.000	0.000	699	0.000	5.88	5.52	–13	–19	–20
lPCgG	0.000	0.000	287	0.000	5.14	4.82	–6	–42	0
lSPL	0.043	0.008	149	0.000	5.09	4.77	–24	–40	47
lCO	0.000	0.000	488	0.000	4.37	4.16	–38	2	10
rOCP	0.014	0.004	179	0.000	3.91	3.76	22	–99	–13
rEnt	0.037	0.008	153	0.000	3.86	3.73	15	6	–27

The table presents the clusters demonstrating significant group differences in R2* values at the cluster level.

*FDR-corr,* *fals- discovery rate-corrected,* *FWE-corr* family-wise error-corrected, *HCs* healthy controls, *lCO* left central operculum, *lCE* left cerebellum exterior, *lMPoG* left posterior gyrus medial segment, *lPCgG* left posterior cingulate gyrus, *lSMC* left supplementary area, *lSMG* left supramarginal gyrus, *lSPL* left superior parietal lobule, *lVD* left ventral diencephalon, *K*_*E*_ cluster extent, *PwPD* patients with Parkinson’s disease, R2* effective transverse relaxation rate, *rAmygdala* right amygdala, *rEnt* right entorhinal area, *rMCgG* right middle cingulate gyrus, *rOCP* right occipital pole, *rPCgG* right posterior cingulate gyrus, *rSFG* right superior frontal gyrus, *Uncorr* uncorrected, *Z*_*E*_ equivalent *z*-score.

**Table 4:** Summary of association analysis between MPM-derived modalities and clinical variables in PwPD

Region (cluster)	*β/r*	SE	*t*	*p*-value	R^2^ with other predictors	VIF	R^2^	R^2^_adj_	RMSE
Multiple linear regression—R2* and LEDD									
lSMA	−5.24	17.45	−0.301	0.766	0.54	2.18	0.33	0.13	272.72
lMPoG	11.75	19.75	0.595	0.557	0.56	2.32			
rMCgG	−89.78	55.78	−1.609	0.121	0.70	3.40			
*rSFG*	*157.83*	*71.26*	*2.215*	*0.037*	*0.59*	*2.49*			
lVD	−12.17	18.61	−0.654	0.520	0.24	1.32			
lCE	7.24	38.55	0.188	0.853	0.28	1.40			
Multiple Linear Regression—R2* and MoCA									
rPCgG	0.17	0.14	1.20	0.241	0.36	1.57	0.38	0.25	1.77
rMCgG	−0.12	0.35	−0.36	0.715	0.49	1.97			
*lMPoG*	*0.63*	*0.28*	*2.23*	*0.034*	*0.35*	*1.48*			
lSMG	0.29	0.35	0.82	0.419	0.27	1.38			
rAmygdala	−0.00	0.11	−0.02	0.983	0.43	1.77			
**Multiple Linear Regression—MTsat and MDS-UPDRS-III**									
*lSFG*	−*14.24*	*6.66*	−*2.13*	*0.042*	*0.14*	*1.16*	0.25	0.13	13.15
lMCgG	−9.58	6.89	−1.39	0.176	0.00	1.00			
CVL I-V	8.95	12.12	0.73	0.467	0.15	1.17			
rCE	−16.75	11.42	−1.46	0.155	0.01	1.01			
**Pearson’s correlation—Proton density and MoCA**									
lSPL	−0.442			0.021					

The table lists the results from multiple linear regression and Pearson’s correlation analyses to probe the association between identified group differences across MPM modalities and clinical presentation in PwPD. Multiple linear regressions were performed with age and sex included as covariates.

*β* β coefficient, *CVL I-V* cerebellar vermal lobules I-V, *lCE* left cerebellum exterior, *LEDD* levodopa-equivalent daily dose, *lMCgG* left middle cingulate gyrus, *lMPoG* left posterior gyrus medial segment, *lPCgG* left posterior cingulate gyrus, *lSFG* left superior frontal gyrus, *lSMA* left supplementary motor area, *lSMG* left supramarginal gyrus, *lSPL* left superior parietal lobule, *lVD* left ventral diencephalon, *MDS-UPDRS-III* Movement Disorders Society’s Unified Parkinson’s Rating Scale (subscore III), *MoCA* Montreal Cognitive Assessment, *MPM* multiparametric mapping, *MTsat* magnetic transfer saturation, *PwPD* patients with Parkinson’s disease, *r* correlation coefficient, *R*^*2*^ coefficient of determination, *R*^2^_*adj*_ adjusted coefficient of determination, R2* effective transverse relaxation rate, *rAmygdala* right amygdala, *rCE* right cerebellum exterior, *rMCgG* right middle cingulate gyrus, *RMSE* root mean squared error, *rPCgG* right posterior cingulate gyrus, *rSFG* right superior frontal gyrus, *SE* standard error, *t* t-statistic, *VIF* variance inflation factor.

### Altered proton density values in the parietal cortex are associated with cognitive state in PwPD

**Fig. 3:**
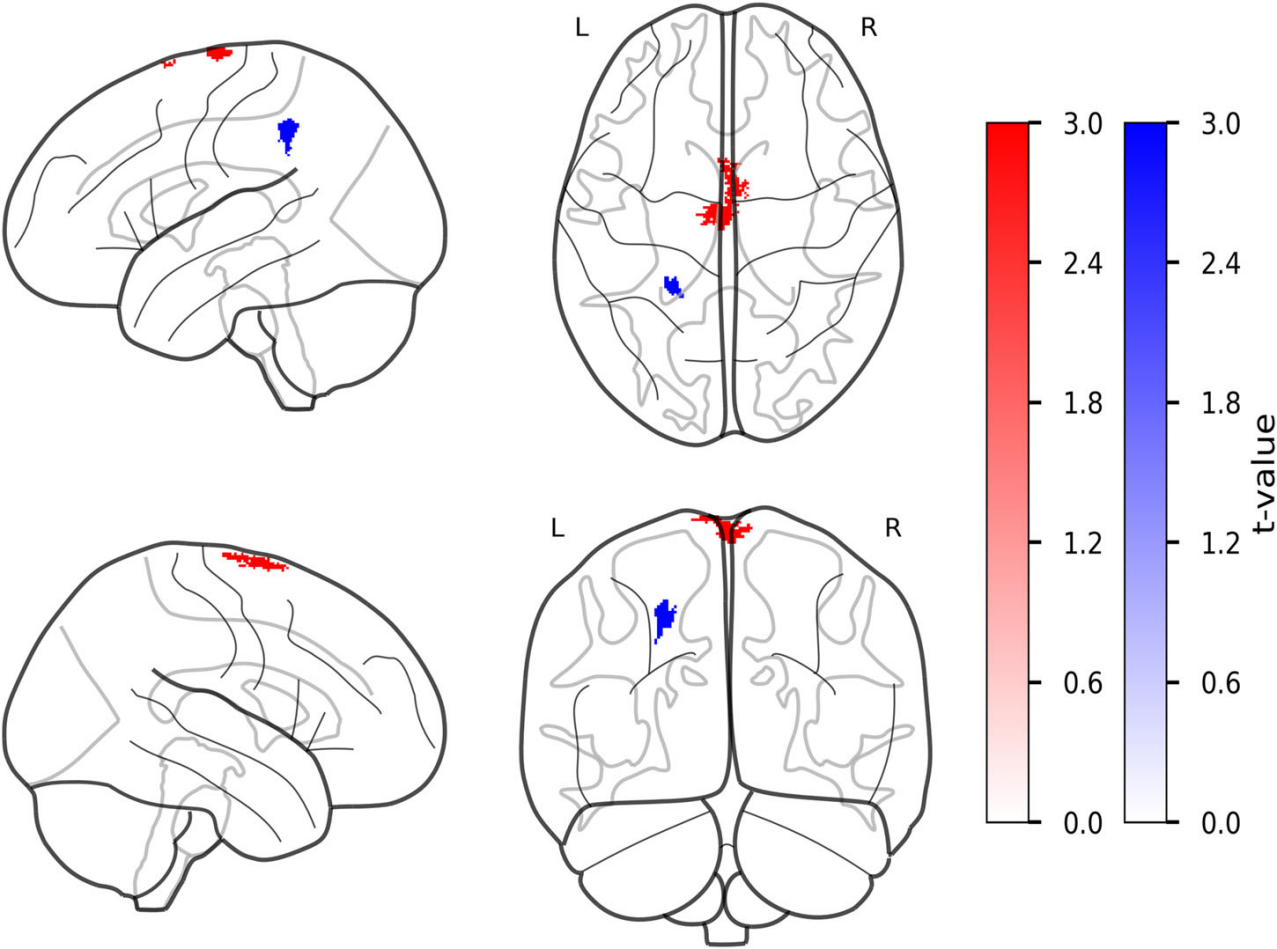
Group differences in proton density values between PwPD and HCs in gray matter. The statistical parametric maps from voxel-wise group comparison of proton density values are presented, displayed on a standard brain template in sagittal, axial, and coronal views. The statistical maps show t-statistics, where red clusters have higher proton density values in PwPD (PwPD > HCs), and blue clusters have higher proton density values in HCs (HCs > PwPD). The color bars reflect voxel-wise t-statistics, with higher absolute *t*-values indicating stronger group differences only. Cluster-defining threshold was set to *T* = 3.18 (*p* < 0.001, uncorrected) with a cluster extent threshold of *k* ≥ 20 voxels. FWE correction was applied at the cluster levels for PwPD > HCs contrast: FWEc = 171, df = [1,94] and HCs > PwPD contrast: FWEc = 157, df = [1,94]. df degrees of freedom, FWE family-wise error, HCs healthy controls, k number of voxels, L left hemisphere, PwPD patients with Parkinson’s disease, R right hemisphere, T t-statistic.

During proton density analysis, we identified significantly higher proton density values in the left supplementary motor area (lSMA) in PwPD (FWEc = 314, *k* = 314, *T* = 5.99 p_FWE_ = 0.000), whereas HCs presented with higher proton density values in the left superior parietal lobule (lSPL, FWEc = 155, *k* = 155, *T* = 4.83, p_FWE_ = 0.026, see Fig. 3 and Table 5). We also performed a Spearman’s correlation test to assess the association between proton density values in the lSPL and MoCA scores, identifying a negative association, suggesting that higher proton density values in lSPL were associated with lower MoCA scores, thus higher cognitive impairment in the PwPD group (*r* = −0.442, *p* = 0.021, 95% CI, −0.70 to −0.07, see Table 4).

**Table 5:** Clusters with significant differences in proton density values between PwPD and HCs

	Cluster level				Peak level		mm	mm	mm
	*P*_FWE-corr_	*q*_FRD-corr_	*K*_E_	*P*_uncorr_	*T*	(*Z*_E_)			
**PwPD** > **HCs**									
lSMA	0.000	0.000	314	0.000	5.99	5.51	0	−11	76
**HCs** > **PwPD**									
lSPL	0.026	0.034	155	0.000	5.16	4.83	−24	−40	47

The table presents the clusters demonstrating significant group differences in proton density values at the cluster level.

*FDR-corr* false-discovery rate-corrected, *FWE-corr* family-wise error-corrected, *HCs* healthy controls, *lSMA* left supplementary motor area, *lSPL* left superior parietal lobule, *K*_*E*_ cluster extent, *PwPD* patients with Parkinson’s disease, *Uncorr* uncorrected, *Z*_*E*_ equivalent *z*-scores.

### Changes in MTsat values in the frontal cortex are linked to motor severity in PwPD

**Fig. 4:**
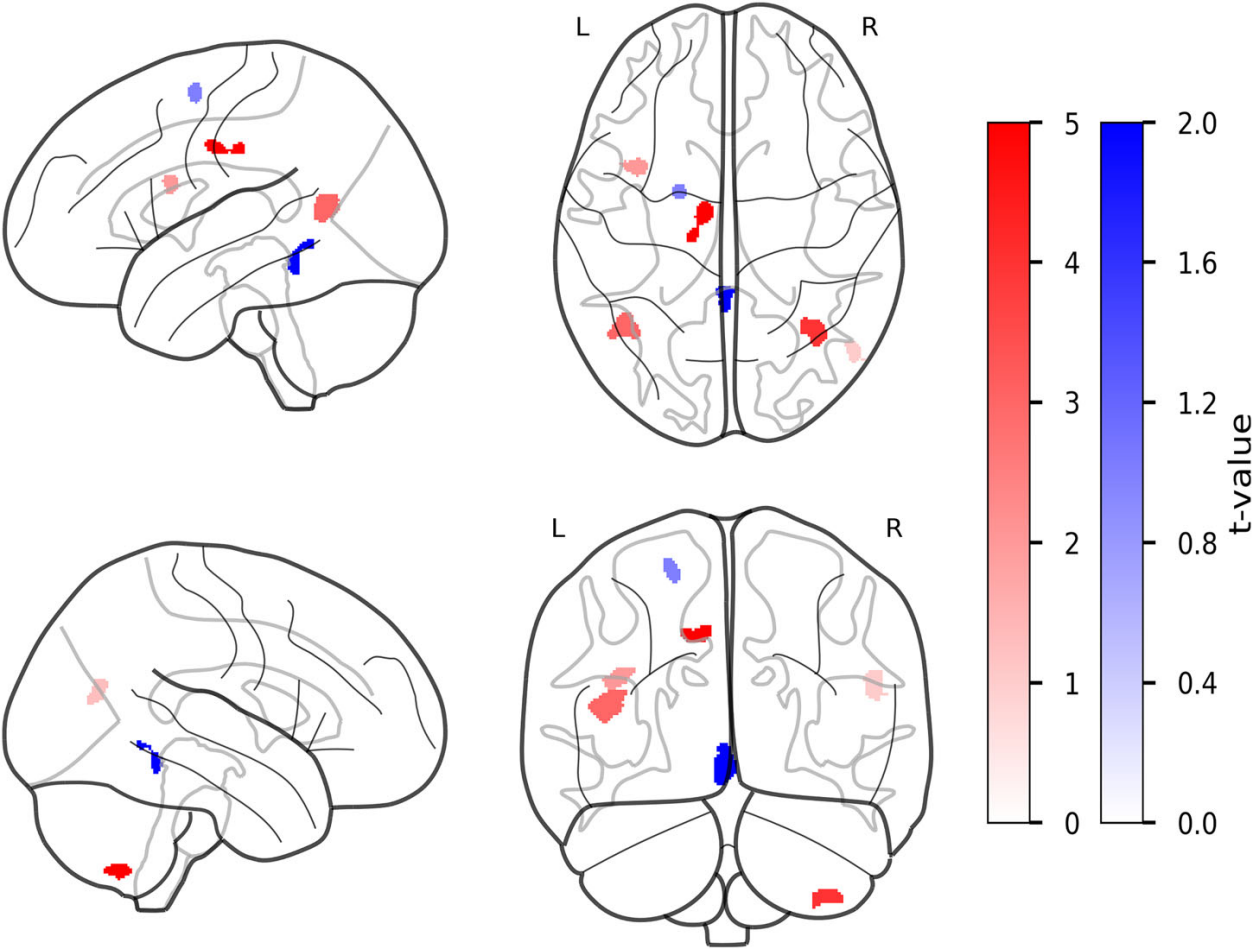
Group differences in MTsat values between PwPD and HCs in gray matter. The statistical parametric maps from voxel-wise group comparison of MTsat values are presented, displayed on a standard brain template in sagittal, axial, and coronal views. The statistical maps show t-statistics, where red clusters have higher MTsat values in PwPD (PwPD > HCs), and blue clusters have higher MTsat values in HCs (HCs > PwPD). The color bars reflect voxel-wise t-statistics, with higher absolute *t*-values indicating stronger group differences only. Cluster-defining threshold was set to *T* = 3.18 (*p* < 0.001, uncorrected) with a cluster extent threshold of *k* ≥ 20 voxels. FWE correction was applied at the cluster levels for PwPD > HCs contrast: FWEc = 231, df = [1,94] and HCs > PwPD contrast: FWEc = 157, df = [1,94]. df degrees of freedom, FWE family-wise error, HCs healthy controls, k number of voxels, L left hemisphere, MTsat magnetic transfer saturation, PwPD patients with Parkinson’s disease, R right hemisphere, T t-statistic.

We identified several clusters with significant group differences in MTsat values for both contrasts (PwPD > HCs: FWEc = 289 and HCs > PwPD: FWEc = 159, see Fig. 4 and Table 6). Clusters in the right CE (rCE, *k* = 332, *T* = 4.09, p_FWE_ = 0.000) and lMCgG (*k* = 360, *T* = 3.91, p_FWE_ = 0.000), among others, demonstrated higher MTsat values in PwPD participants. While MTsat values in the left SFG (lSFG, *k* = 154, *T* = 4.42, p_FWE_ = 0.040) and cerebellar vermal lobules I-V (CLV I-V, *k* = 307, *T* = 3.72, p_FWE_ = 0.000, see Fig. 4 and Table 6) were higher in HCs. Following group comparison, we extracted mean MTsat values from lSFG, CVL I-V, rCE, and lMCgG, using them as predictors for a multiple regression model to assess their association with motor symptom severity among the PwPD group informed by MDS-UPDRS-III scores. Only the lSFG MTsat values significantly predicted MDS-UPDRS-III scores, suggesting that lower MTsat values within this cluster in lSFG were associated with higher MDS-UPDRS-III scores, subsequently greater motor severity in PwPD.

**Table 6:** Clusters with significant differences in MTsat values between PwPD and HCs

	Cluster level				Peak level		mm	mm	mm
	*P*_FWE-corr_	*q*_FRD-corr_	*K*_E_	*P*_*uncorr*_	*T*	*(Z*_*E*_*)*			
**PwPD** > **HCs**									
lMTG	0.000	0.000	613	0.000	5.65	5.24	−41	−59	14
rMGO	0.000	0.000	310	0.000	4.12	3.94	53	−69	−19
rCE	0.000	0.000	332	0.000	4.07	3.90	37	−60	−54
lOpIFG	0.000	0.000	289	0.000	4.00	3.83	−35	6	23
lMCgG	0.000	0.000	360	0.000	3.91	3.76	−9	−11	38
**HCs** > **PwPD**									
lSFG	0.044	0.022	154	0.000	4.42	4.20	−19	−3	61
CVL I-V	0.000	0.000	307	0.000	3.87	3.72	−1	−44	−7

The table presents the clusters demonstrating significant group differences in MTsat values at the cluster level.

*CVL I-V* cerebellar vermal lobules I-V, *FDR-corr* false-discovery rate-corrected, *FWE-corr* family-wise error-corrected, *HCs* healthy controls, *lMCgG* left middle cingulate gyrus, *lMTG* left middle temporal gyrus, *lOpIFG* left opercular part of the inferior frontal gyrus, *lSFG* left superior frontal gyrus, *K*_*E*_ cluster extent, *MTsat* magnetic saturation transfer, *PwPD* patients with Parkinson’s disease, *rCE* right cerebellum exterior, *rMGO* right middle occipital gyrus, *Uncorr* uncorrected, *Z*_*E*_ equivalent *z*-scores.

### Observed group differences in some brain clusters overlap across multiple MPM modalities

**Fig. 5:**
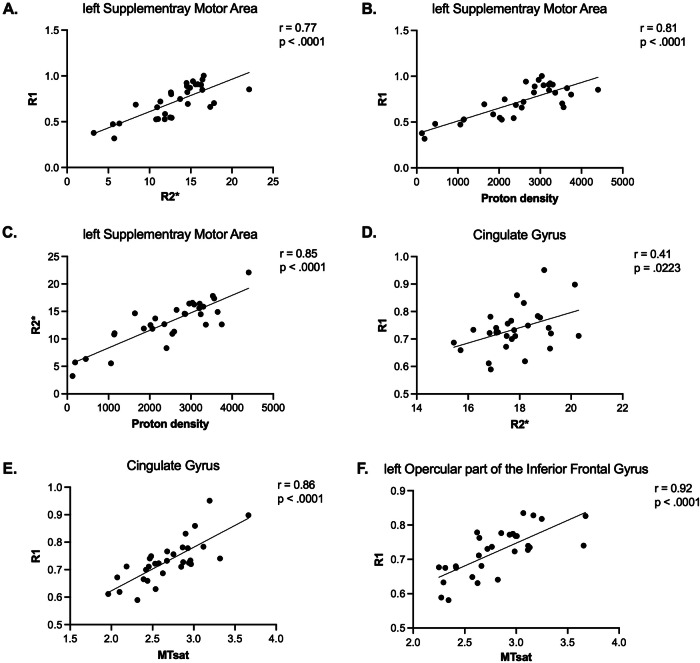
Scatter plots of cross-modal correlation between mean MPM parameter values in clusters with significant group differences in gray matter. The figure presents multiple panels with scatter plots and key statistical data assessing the correlation between mean MPM-derived parameters from overlapping clusters with significant differences between PwPD and HCs in the left supplementary motor area (**A**−**C**), cingulate gyrus (**D**, **E**), and left opercular part of the inferior frontal gyrus (**F**). Only the clusters that passed the Bonferroni correction (*p* < 0.00625) were included in the presented analysis. HCs healthy controls, MPM multiparametric mapping, MTsat magnetic transfer saturation, PwPD patients with Parkinson’s disease, R1 longitudinal relaxation rate, R2* effective transverse relaxation rate.

**Fig. 6:**
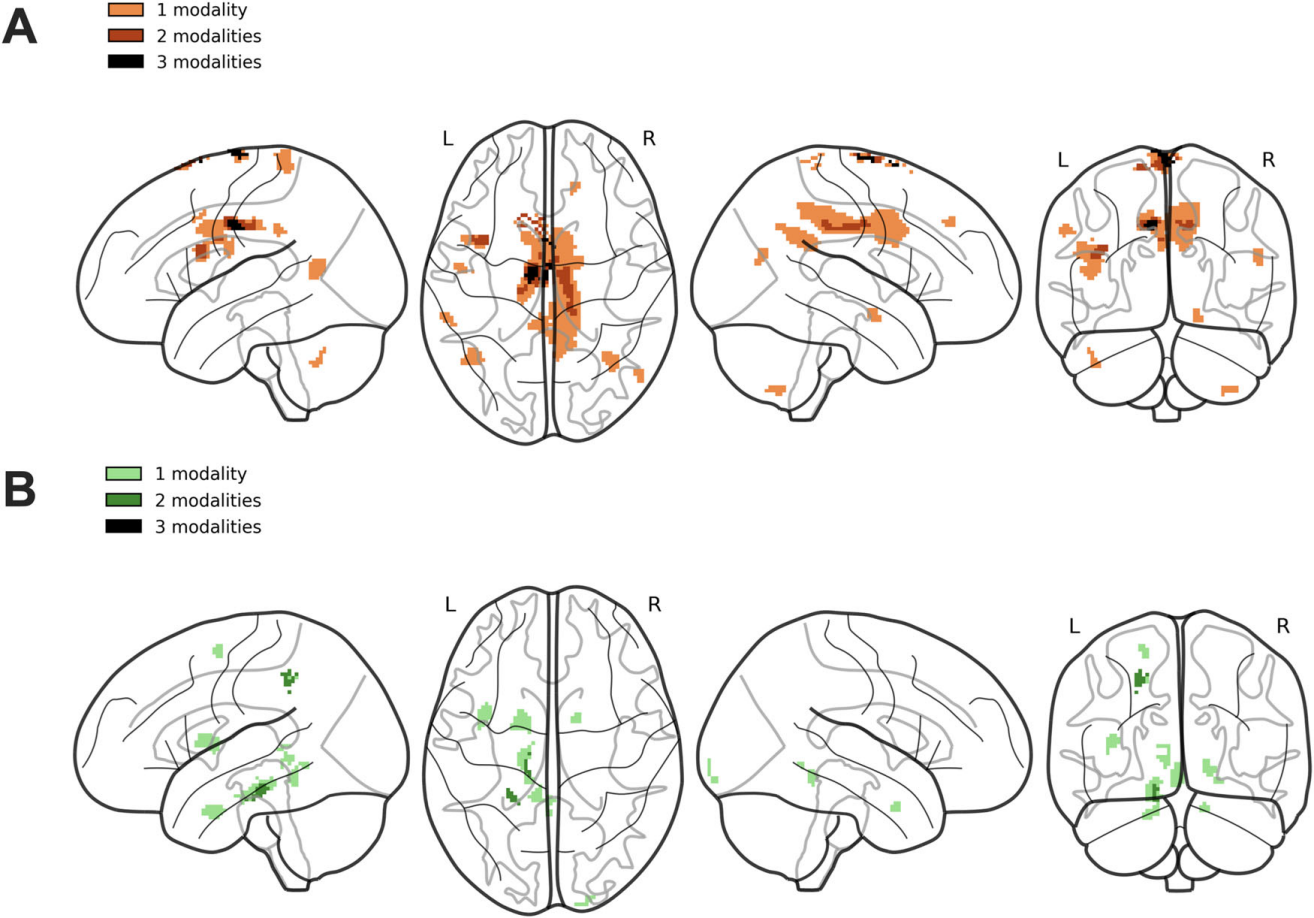
Venn-encoded overlap of identified group differences across MPM modalities in gray matter. The figure illustrates the clusters with identified significant group-based differences in biophysical properties informed by MPM modalities, including R1, R2*, proton density, and MTsat. Identified clusters are color-coded depending on the number of MPM modalities with identified differences. **A** Identified clusters had mean values across MPM modalities that are higher in PwPD; consequently, **B** Presented clusters with higher mean values in HCs. HCs healthy controls, L left hemisphere, MPM multiparametric mapping, MTsat magnetic transfer saturation, PwPD patients with Parkinson’s disease, R right hemisphere, R1 longitudinal relaxation rate, R2* effective transverse relaxation rate.

For the cross-modal correlation, we used only clusters that survived an additional Bonferroni correction (*p* < 0.00625). Higher R1 values colocalized with observed higher R2* in the lSMA and cingulate cortex (CgG), which were confirmed to have a positive correlation (lSMA: *r* = 0.77, *p* < 0.0001; CgG: *r* = 0.41, *p* = 0.0223, see Figs. 5 and 6). In addition, higher R1 in PwPD positively correlated with higher MTsat in CgG (*r* = 0.86, *p* < 0.0001) and the opercular part of the left inferior frontal gyrus (lOpIFG, *r* = 0.92, *p* < 0.0001). In addition, we identified a positive correlation between R2* and proton density values in lSMA (*r* = 0.85, *p* < 0.0001).

### VBQ analysis of the basal ganglia and substantia nigra revealed no significant group-level differences

Using the ATAG atlas, we create the basal ganglia (BG) region-of-interest (ROI) R1, R2*, proton density, and MTsat maps^20^. We observed no significant group differences across any MPM-derived modality in the BG. To understand whether the lack of findings in the BG is due to the MPM methodological limitation, we performed ROI-based VBQ analysis in the SN. Similarly to the BG, we used the ATAG atlas to create a SN mask, used to derive ROI-based R1, R2*, proton density, and MTsat maps^20^. For ROI-based VBQ analysis in the SN, we used quantitative maps with different levels of spatial smoothing (6 × 6 × 6 mm FWHM and 4 × 4 × 4 mm FWHM) and unsmoothed maps to ensure that potential group differences are not obscured by spatial smoothing, as the SN is a smaller structure and can be disproportionately affected by the preprocessing procedure. Across all differently processed quantitative maps, we detected no significant group differences across MPM modalities in the SN.

### MPM analysis revealed widespread changes in PwPD in surrogate markers of white matter microstructure

Utilizing the same approach for WM analysis, we observed widespread microstructural changes in PwPD when compared to HCs. Remarkably, all of the identified clusters with higher R1, R2*, proton density, and MTsat values in PwPD were anatomically close to clusters identified with significant group differences during GM analysis, indicating spatially overlapping microstructural changes across different types of brain matter. WM clusters with identified higher R1, R2*, proton density, and MTsat values were statistically significant. (R1: *T* = 3.18, PwPD > HCs: FWEc = 137; R2*: *T* = 3.18, PwPD > HCs: FWEc = 129, HCs > PwPD: FWEc = 142; proton density: *T* = 3.18, PwPD > HCs: FWEc = 122; MTsat: *T* = 3.18, PwPD > HCs: FWEc = 167, HCs > PwPD: FWEc = 138, see Supplementary Figs. 2-5 and Supplementary Tables 1-4). In comparison to GM, a small number of clusters were identified to have higher R2* and MTsat values in HCs (*n* = 3 and 7, respectively), with no clusters identified for R1 or proton density modalities for this contrast. Similar to GM findings, numerous clusters with identified group differences, presented with changes across multiple MPM modalities (see Supplementary Figs. 6-8).

## Discussion

In this study, we utilized MPM to identify cortical microstructural alterations characteristic of PwPD compared to HCs. Our findings showed distinct spatial patterns across all four MPM-derived metrics, reflecting various underlying PD-associated pathological processes. The main advantage of MPM is its ability to probe a wide spectrum of disease processes underlying microstructural changes, such as neuroinflammation, demyelination, and iron accumulation, while enabling tissue-specific analysis based on explicit masking of GM and WM, all within a single acquisition period, directly in PwPD with minimum patient burden. As MPM-derived parameters are biologically grounded, the observed distinct spatial group differences are reflective of specific biological processes underlying the observed microstructural change, providing insights beyond the traditional structural neuroimaging. Furthermore, as some of the MPM-derived modalities are partially sensitive to overlapping tissue properties, such as iron and myelin content, cross-modal validation within MPM modalities would strengthen interpretability, especially when consistent changes are observed across the same clusters.

We consistently identified group differences across multiple modalities, frequently overlapping in the same clusters located in the cingulate cortex, cerebellum, and frontal regions, suggesting that overlapping microstructural abnormalities result from multifactorial pathology. Observed R2* alterations frequently accompanied changes in R1 and MTsat, indicating that iron changes are linked with myelin disruptions or tissue remodeling. For instance, increased R2*, R1, and proton density values in the lSMA may indicate neuronal loss, gliosis, and iron accumulation. In addition, we identified higher R1, R2*, and MTsat in the cingulate cortex, higher R2* in rSFG, and higher R1 and MTsat in the OpIFG and MTG. Our observations are consistent with transcriptomic studies demonstrating dysregulations in the cingulate cortex in PwPD, suggesting that higher R1 and MTsat may reflect pathogenic events, rather than compensatory remyelination^21^. As R1 and MTsat are both sensitive to macromolecular and myelin content, the higher values that we observed in the OpIFG and MTG may reflect compensatory remyelination and tissue remodeling rather than primary disease mechanisms, as we observed no associations with MoCA scores.

In addition to MPM-derived parameter group differences, we observed an association between identified group differences and clinical parameters. For instance, an identified positive correlation between R2* in PCgG and MoCA scores in PwPD, supported by existing literature. The cingulate cortex is involved in emotion regulation and decision-making, and other studies demonstrate that metabolism in the MCgG is negatively associated with impaired self-awareness, and that an impaired PCgG metabolism is a risk factor for mild cognitive impairment in PwPD^22,23^. Furthermore, a previous study demonstrated a link between cortical thinning of the IFG and impulse control disorder in PwPD, implicating this brain area in the disease’s clinical presentation^24^. lSMA plays a role in motor planning and execution; however, we observed no association between iron buildup in lSMA and motor symptom severity or disease duration, suggesting that a combination of microstructural changes across multiple brain regions may underlie observed PwPD symptomatology, rather than alterations within a single cluster. Additionally, higher R2* in SMA has been linked to greater depressive symptoms and olfactory dysfunction in PwPD in other studies, symptoms not directly related to SMA function, supporting the idea that widespread microstructural alterations rather than isolated changes underlie PwPD symptomatology^25^. The identified higher R2* in rSFG positively correlated with LEDD in PwPD. Since higher LEDD indicates more advanced motor symptoms, higher pharmacological needs associated with increased iron in the rSFG, involved in voluntary motor planning and execution, may further support the biological relevance of the derived MPM data^25,26^.

Additionally, we observed a lowering of MPM values in PwPD, reflecting neurodegenerative processes, such as demyelination (lower R1 and MTsat), reduced iron content or neuronal loss (lower R2*), and reduced intracellular water content (lower proton density), all consistent with microstructural disruptions. Therefore, the detected lower MTsat in bilateral SFG demonstrates ongoing demyelination and neuronal atrophy, while lower R2* and proton density in lSPL in PwPD reflect reduced water content and iron deposition, in line with neuronal atrophy. Our findings align with existing research showing that neuronal atrophy in PwPD, presented as lower MTsat, is further supported by studies demonstrating that lower local gyrification index across multiple brain regions, including the SFG, posterior, and cingulate areas, helps differentiate PwPD with affective symptoms such as depression from those without^27,28^. While parietal lobe atrophy has been reported as a marker of PD progression in moderate-to-late stages, we did not replicate this, possibly due to differences in cohort disease severity^17^

Similarly, our findings demonstrated that lower MPM values in specific brain areas are associated with clinical measures, such as lower MTsat in SFG and MDS-UPDRS-III. This observation aligns with existing research showing that altered synchronization within the SFG is associated with motor severity in PwPD, such as dyskinesia, highlighting the SFG’s prominent role in PD motor symptoms^29^. Additionally, we found a negative correlation between SPL proton density and MoCA scores in PwPD, reflecting SPL involvement in visuospatial and cognitive integration of sensory and executive processes.

The derived findings are in line with available literature, supported by studies demonstrating a reduction in structural integrity in the bilateral temporal, parietal, and occipital lobes in moderate-to-late stage PwPD supports this^17^. Although we did not observe correlation with disease duration, possibly due to our cohort mainly comprising of early-stage PwPD (mean disease duration: 4.52 ± 4.31 years), another study reported progressive atrophy in multiple regions, including the left superior and right temporal gyri, superior frontal, cingulate gyri, occipital lobe, and bilateral cerebellum as PD progresses^30^. The observed group differences in these regions further highlight the need for additional longitudinal studies to assess MPM’s potential for tracking disease progression. The detection of compensatory changes in the IFG, MTG, and right occipital pole is supported by evidence of negative associations between cortical thinning in these brain regions and memory performance in PwPD, which are believed to precede macrostructural changes and the development of cognitive impairment^31^. Moreover, reduced GM volume in the bilateral superior, middle temporal, and right middle occipital gyri has been linked to impulse control disorder in PwPD^32^. However, we did not observe neuronal atrophy in these brain regions, possibly due to the cohort’s disease severity.

PD is traditionally associated with GM loss in the SN and basal ganglia; nevertheless, WM degeneration is increasingly recognized as a contributing factor to disease progression. Research shows that WM loss precedes the GM loss and independently influences motor symptom severity, with growing evidence of ongoing WM degeneration and demyelination as PD progresses^19,33,34^. Diffusion tensor imaging (DTI) is the standard method for assessing WM integrity by measuring water diffusion across tissue. However, DTI has limited spatial resolution compared to conventional MRI and lower specificity due to confounding factors^35^. Using MPM, we examined WM microstructure, finding widespread changes in PwPD, such as co-localized higher R1, R2*, and proton density values in the same clusters. As mentioned, higher R1, R2*, and proton density can be interpreted in several ways, including remyelination or gliosis and neuroinflammatory processes^36,37^. Given the multifactorial interpretation of observed changes, some observed microstructural changes may have resulted from compensatory remyelination, which is well-documented in structural reorganization in PD^19,38–42^. Nonetheless, understanding the full extent and disentangling overlapping processes will require future studies utilizing multimodal approaches such as a combination of DTI and MPM in the same cohort for validation and more precise interpretation.

Despite successfully identifying known microstructural changes in PwPD, our study has limitations, such as a selective bias toward early and moderate PD stages. Imaging of advanced and late-stage PwPD is challenging due to more severe motor symptoms resulting in higher motion artifacts, as well as increased patient burden. However, a 30-minute acquisition protocol remains susceptible to motion artifacts, subsequently affecting derived MPM maps. Bearing this limitation in mind, we adapted rigorous, two-step quality control, but the impact of artifacts cannot be completely ruled out. Additionally, the precision of MPM-derived quantitative maps is highly sensitive to variations in scanner hardware, sequence parameters, and calibration methods, necessitating rigorous standardization to ensure cross-subject comparability and data reproducibility^8^. Our study was limited to the use of MoCA, which reflects global cognition, necessitating the use of a comprehensive neuropsychological battery to characterize domain-specific cognitive impairments in PwPD in future studies.

Intrestingly, we did not replicate the commonly documented iron accumulation within BG nuclei in the PwPD^43–45^. However, several studies reported no significant differences in iron content within the BG in PwPD and HCs^46–48^. This discrepancy may be explained by the disease of the investigated cohorts: both in our study and in studies reporting no differences, the cohorts comprised early- to moderate-stage PwPD, suggesting that BG iron accumulation is disease stage-dependent. This idea is further confirmed by our ROI-based analysis in the SN, demonstrating that the null findings in the BG and SN reflect the lack of significant difference in microstructure between PwPD and HCs, rather than the limitation of MPM, such as VBQ spatial smoothing or voxel-based interference. Furthermore, findings from longitudinal research suggest that iron accumulation progression is a more reliable clinical biomarker when compared to static group differences at a given stage^49,50^. Interestingly, various BG nuclei follow various time-dependent trajectories of iron composition^44^. For instance, SN and globus pallidus are reported to have significant group differences at later stages of the disease, while the putamen and red nucleus group differences are more pronounced in early, but not late stages of PD^44^. In support of these findings, one study utilized MPM, detecting significant differences in the iron accumulation in the putamen where iron gradients across the anatomical region were quantified^51^. As our study comprised PwPD with comparable disease severity, we quantified the mean MPM parameter across regions rather than investigating spatial gradients, complicating direct comparison. Additional consideration lies in the technique used for iron content assessment; most of the previous studies used quantitative susceptibility mapping (QSM), while we used R2* values. As previously stated, R2* values are affected by various biological entities, including myelin, complicating reliable separation of their contribution at 3 T and even 7 T^52^. MRI followed by histopathology further supports the idea of interpreting R2* values as iron content is misleading, and this readout should be considered as a marker of general microstructural integrity instead^53^. Moreover, MPM has a limited spatial resolution for the reliable assessment of small subcortical structures such as SN, which is a critical limitation of the current MPM protocols. Nevertheless, having demonstrated the feasibility and utility of implementing MPM, further research should account for these limitations to improve its interpretability and reproducibility.

MPM’s ability to identify PD-related microstructural changes and their association with disease progression highlights its potential for broader research applications. For example, histopathological validation through post-mortem studies can confirm the biological basis of MPM findings, such as gliosis and iron accumulation^54,55^. MPM can also be combined with molecular or fluid biomarkers such as cerebrospinal fluid (CSF) or PET indicators of neuroinflammation. Post-mortem studies and gene expression atlases can reveal the cellular and chemical compositions of underlying tissue changes. One study demonstrated that microstructural changes correlated with regional transcriptomic signatures, particularly with PD-related genes^56^. Cingulate, frontal, and temporal lobes were identified to be especially vulnerable to microstructural disruptions, even prior to motor decline or therapeutic intervention, further supporting our findings^56^. Similarly, changes in the frontal and cingulate cortices were associated with oligodendrocyte and oligodendrocyte-precursor cell expression, confirmed by post-mortem data, offering insights into demyelination, remyelination, compensatory capacities, and helping to differentiate pathogenic and compensatory changes during the PD course^57^. Since our study was performed at a single time point, future longitudinal research is needed to determine whether microstructural changes precede clinical presentation and the progression of the microstructural changes along the disease course. Additionally, MPM could serve as a tool for patient stratification by capturing individual microstructural differences, enabling more personalized treatment strategies that target affected brain regions, align with specific clinical symptoms, reduce side effects, and improve efficacy.

To conclude, our study illustrates MPM’s value and biological interpretability across whole-brain analysis. Multiple converging findings highlight the multifactorial nature of PD pathology, involving demyelination, neuroinflammation, gliosis, and iron accumulation, underscoring disease complexity. Therefore, MPM holds significant potential as an accessible, cost-effective in vivo method to directly assess microstructural changes with minimal patient burden and high interpretability. It holds great promise for ongoing disease monitoring, patient stratification, and tailored therapeutic development.

## Methods

### Experimental design and participants

Participants were recruited from the inpatient and outpatient clinics at the Department of Neurology of the University Medical Center Schleswig-Holstein (Campus Lübeck) and provided written informed consent. Eligible participants were between 50 and 80 years old and had no concomitant neurological or psychiatric condition. PwPD were selected based on a clinically confirmed PD diagnosis using the Movement Disorders Society Clinical Diagnostic Criteria^58^. Exclusion criteria for both groups included comorbidities affecting the ability to provide informed consent, MRI contraindications, diagnosis of atypical or secondary Parkinsonism, history of alcohol abuse, or structural brain diseases (e.g., stroke). All PwPD underwent extensive clinical examinations comprising the MDS-UPDRS-III and MoCA assessments, followed by one MRI session^59,60^. HCs underwent only the MRI session. A total of 114 German-speaking persons were enrolled. The study adhered to the Declaration of Helsinki and was reviewed and approved by the local ethics committee at the University of Lübeck, Germany (AZ: 2024-302_2).

### Image acquisition and voxel-based quantification

MRI sessions were conducted at the Center for Brain, Behavior, and Metabolism at the University of Lübeck, using a 3 Tesla (T) Siemens Magnetom Skyra scanner with a 64-channel head/neck coil. We acquired MPM-derived data using a multi-echo 3D fast low-angle shot (FLASH) sequence, as previously described^13^. Volumes were acquired with T1, proton density, and magnetization transfer (MT) weightings. All sequences were acquired in 3D with 240 contiguous slices, 1.0 mm isotropic resolution, a field of view of 256 × 176 mm² (phase FoV 68.8%), GRAPPA acceleration factor 2, and partial Fourier 6/8 in the slice direction. The receiver bandwidth for all echoes was 440 Hz/px. Inline distortion correction (2D mode) and prescan normalization were enabled. The T1-weighted acquisitions (TR = 19.0 ms, flip angle = 20°, six echoes with TE = 2.20/4.70/7.20/9.70/12.20/15.00 ms) had an acquisition of approximately 8 minutes. The proton density-weighted acquisitions (TR = 24.0 ms, flip angle = 20°, eight echoes with TE = 2.20/4.70/7.20/9.70/12.20/15.00/17.50/20.00 ms) required approximately 11 minutes. The MT-weighted acquisition (TR = 37.0 ms, flip angle = 20°, six echoes with TE = 2.20/4.70/7.20/9.70/12.20/15.00 ms) had an acquisition time of approximately 7 minutes, resulting in a total protocol duration of approximately 26 minutes.

All acquired datasets have been reviewed by an experienced neuroradiologist to exclude concurrent conditions. In addition, visual assessments of artifacts (e.g., extensive head motion) as well as quantitative evaluations based on the calculation of voxel-wise z scores for each MPM-derived parameter, have been performed. We excluded participants with severe visible motion artifacts (ringing and blurring) and excessive quantitative measures, defined as *z*-scores greater than two standard deviations above the mean distribution.

We processed the obtained images using the hMRI toolbox, within the Statistical Parametric Mapping 25 framework (SPM25, Wellcome Trust Center for Neuroimaging, London) in MATLAB software (R2025a, The MathWorks Inc., Natick, MA, USA)^8^. Using a custom-written MATLAB script, we reconfigured the hMRI toolbox with scanner-specific settings to reconstruct quantitative and semi-quantitative maps of R1, R2*, proton density, and MTsat. For quantitative map reconstruction, we used the ‘Create hMRI maps’ function, using our T1-, proton density-, and MT-weightings as input images. We implemented B1 bias correction using the ‘UNICORT’ setting, and radiofrequency sensitivity bias was corrected with a unified segmentation procedure in the hMRI toolbox. Once maps have been created and visually assessed for the quality of the data, we perform preprocessing utilizing the hMRI toolbox pipeline comprising segmentation (into GM, WM, and CSF maps), nonlinear spatial registration to MNI space, and tissue-weighted smoothing^9,61,62^. The MPM-derived maps preprocessing settings were set to a resampled voxel size of 1 × 1 × 1 mm^3^, a bounding box of 2 × 3, and Gaussian smoothing with a 6 × 6 × 6 mm full-width at half maximum (FWHM) kernel.

### Statistical analyses

We conducted cluster-based analyses in SPM25 within MATLAB. The second-level analysis involved a two-sample *t*-test to compare surrogate markers of microstructural integrity (e.g., R1, R2*, proton density, and MTsat) between PwPD and HCs. We included age and sex as nuisance regressors. We set the cluster-forming threshold of *p* < 0.001 (uncorrected), and statistical significance was assessed at *p* < 0.05, cluster-level, FWE-corrected. We tested two contrasts: PwPD > HCs and HCs > PwPD. We extracted clusters with identified significant differences and visualized them on a glass brain using Python software (v3.13.5, Python Software Foundation) and libraries such as Nilearn (v.0.12.0)^63,64^.

We applied multiple regression models to explore relationships between mean R1, R2*, proton density, and MTsat in significant clusters and clinical data, such as MDS-UPDRS-III, LEDD, disease duration, and MoCA scores; the regression analyses were not corrected for multiple corrections. We included age and sex as covariates in multiple linear regression analyses. Using the statsmodels library (v0.14.5) within the Python environment, we performed multiple regression analysis^65^. For multiple linear regression, we selected clusters that demonstrated significant group-level differences and were deemed anatomically and functionally relevant to PwPD clinical presentation. We performed these post-hoc analyses using MarSBaR (v0.45) to extract mean parameter values within the cluster of interest, using them as predictors in the multiple regression models.

For the analysis of cross-modal correlation within clusters with significant group differences, we only selected clusters that survived additional Bonferroni correction across modalities. We performed the statistical analysis for four MPM-derived maps (R1, R2*, proton density, and MTsat) and two contrasts (PwPD > HCs and HCs > PwPD); therefore Bonferroni threshold was calculated as ɑ = 0.05 / 8 tests, resulting in *p* < 0.00625. We extracted mean parameter values using MaSBaR and performed Pearson’s correlation to investigate the relationship between R1, R2*, proton density, and MTsat within the same clusters.

All analyses were performed separately for each quantitative map (R1, R2*, proton density, and MTsat), as well as for GM and WM, using explicit masks where applicable. For the whole-brain analysis, we utilized tissue probability masks incorporated within the SPM25 environment. We set the SPM25 making threshold to ‘none’ without any additional intensity-based exclusion. Significant clusters were anatomically labeled using the Neuromorphometrics atlas for GM integrated within the SPM25 environment and the JHU White-Matter Tractography Atlas for WM^66–68^.

For the VBQ analysis in the BG and SN, we used the ATAG atlas to create BG and SN masks^20^. Using the respective masks, we performed separate analyses for each MPM modality. The VBQ analysis in the SN was repeated twice using quantitative maps with different levels of spatial smoothing, 4 × 4 × 4 mm FWHM, and unsmoothed maps. These maps were preprocessed in the same manner, including segmentation, nonlinear spatial registration to MNI space, and tissue-weighted smoothing, except that the smoothing kernel was reduced from 6 × 6 × 6 mm FWHM to 4 × 4 × 4 mm FWHM and 0 × 0 × 0 mm FWHM.

## Supplementary information

Supplementary Information

## Data Availability

The data supporting this study are available from the corresponding author upon reasonable request. The code used for the analyses in this study is available from the corresponding author upon reasonable request.

## Code availability

The code used for the analyses in this study is available from the corresponding author upon reasonable request.
