# Supplementary material for: In-vivo histology of Parkinson’s disease using quantitative multiparametric mapping
Source: NPJ Parkinsons Dis. 2026 Mar 31;12:82. doi: 10.1038/s41531-026-01329-4 (PMC13046743; doi:10.1038/s41531-026-01329-4)
Supplement: Supplementary file 1 — Supplementary Information [file 41531_2026_1329_MOESM1_ESM.docx]

**SUPPLEMENTARY FIGURES:**

**
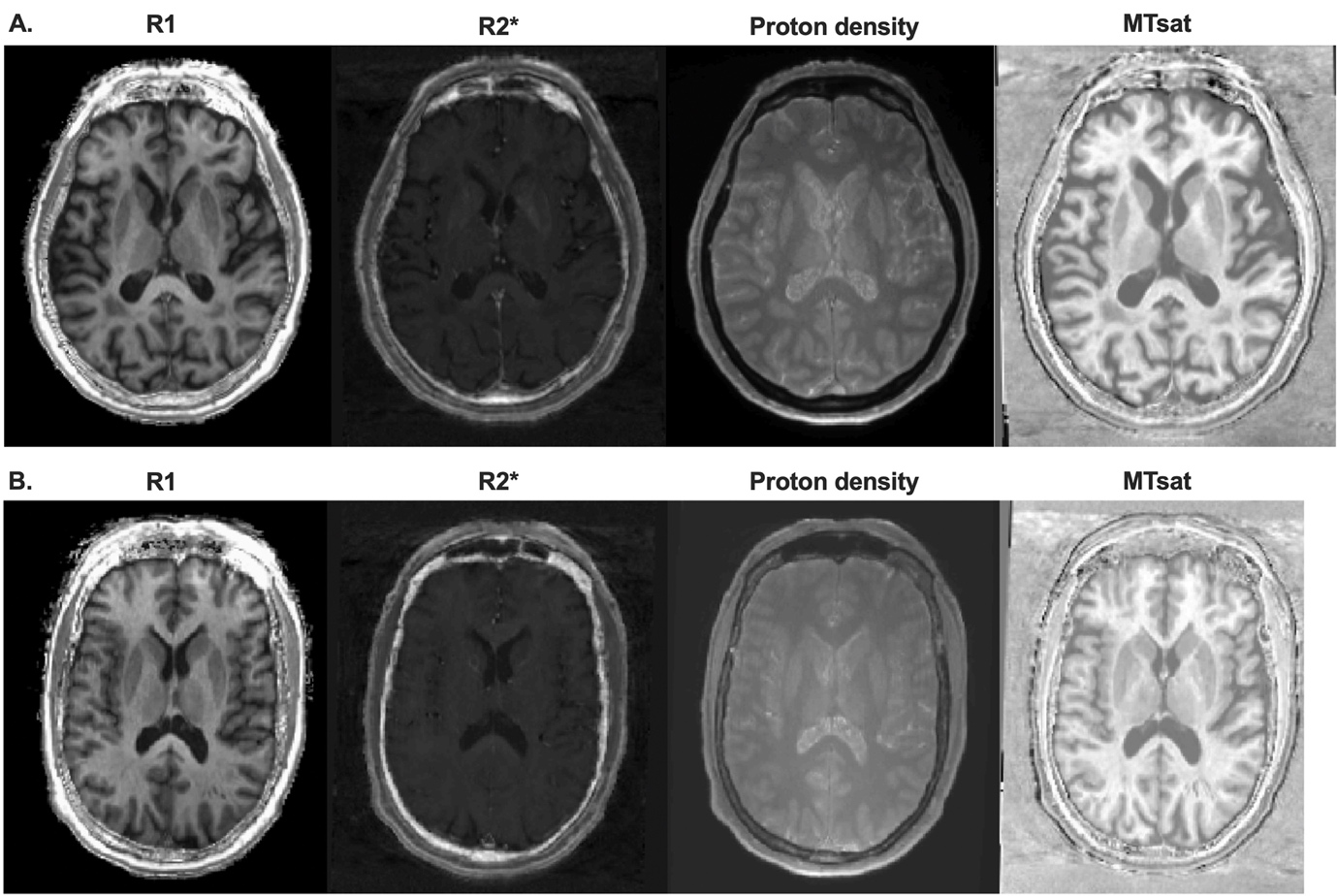
**

**Supplementary Figure 1. Representative unprocessed quantitative parameter maps from participants.** We present axial slices of four raw quantitative MPM-derived maps, R1, R2*, proton density, and MTsat to demonstrate image quality and spatial distribution. A. The images are derived from a healthy 77-year-old female participant in native spaces. B. The images are derived from a 71-year-old female patient with Parkinson’s disease in native spaces. MPM: multiparametric mapping. MTsat: magnetic transfer saturation. R1: longitudinal relaxation rate. R2* effective transfer relaxation rate.


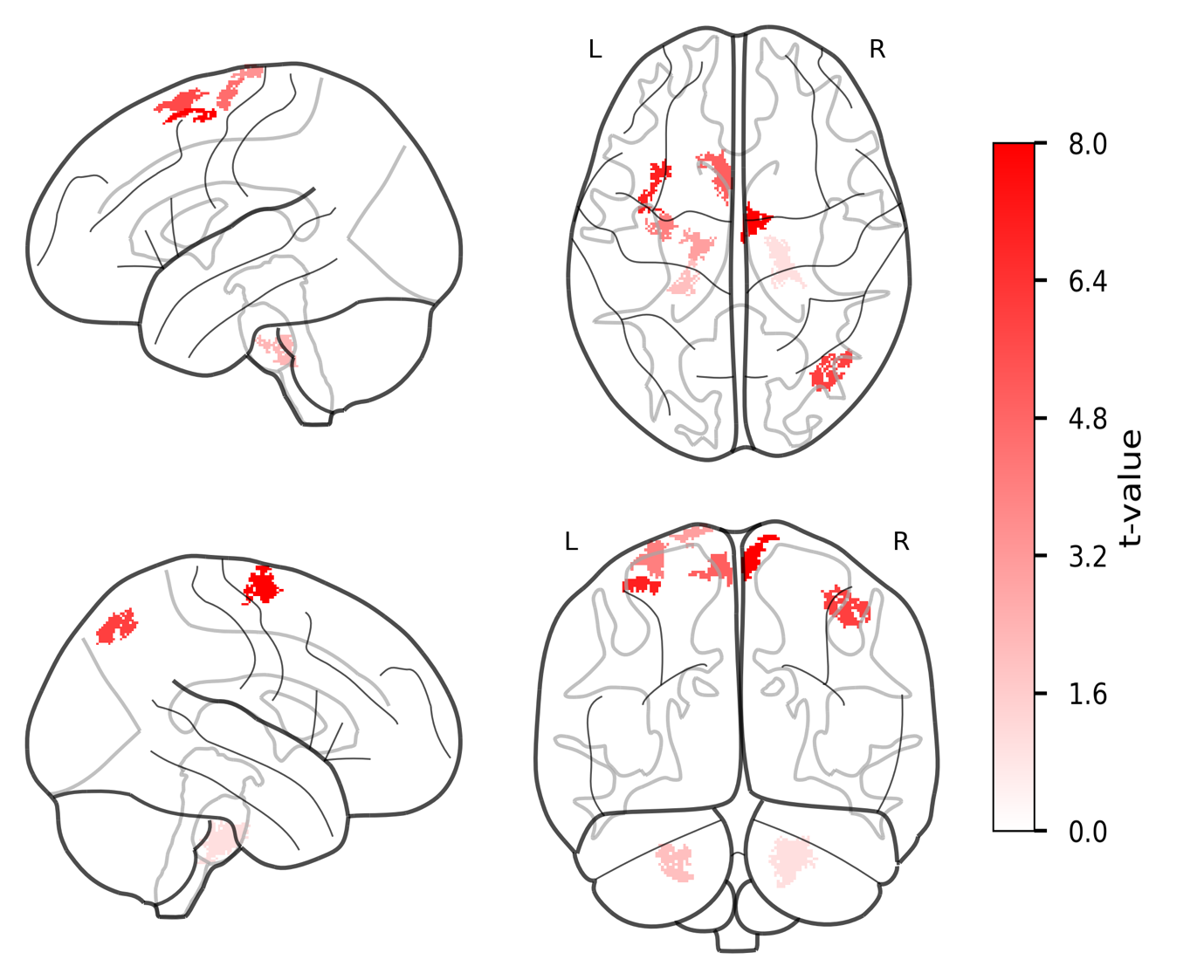


**Supplementary Figure 2. Group differences in R1 values between PwPD and HCs in white matter.** The statistical parametric maps from voxel-wise group comparison of R1 values are presented, displayed on a standard brain template in sagittal, axial, and coronal views. The statistical maps show t-statistics, where red clusters have higher R1 values in PwPD (PwPD > HCs). The color bar reflects voxel-wise t-statistics, with higher absolute t-values indicating stronger group differences only. Cluster-defining threshold was set to T = 3.18 (p < .001, uncorrected) with a cluster extent threshold of k ≥ 20 voxels. FWE correction was applied at the cluster levels for the PwPD > HCs contrast: FWEc = 137, df = [1, 94]. df: degrees of freedom. FWE: family-wise error. HCs: healthy controls. k: number of voxels. L: left hemisphere. PwPD: patients with Parkinson’s disease. R: right hemisphere. R1: longitudinal relaxation rate. T: t-statistic.

**Supplementary Table 1. Clusters with significant differences in R1 values between PwPD and HCs in white matter.**

|  | *cluster level* | | | | *peak level* | | *mm* | *mm* | *mm* |
| --- | --- | --- | --- | --- | --- | --- | --- | --- | --- |
|  | *P*_FWE-corr_ | *q*_FRD-corr_ | *K*_E_ | *P*_uncorr_ | *T* | (*Z*_E_) |  |  |  |
| **PwPD > HCs** | | | | | | | | | |
| MCP | .000 | .000 | 328 | .000 | 5.80 | 5.36 | 22 | -24 | -35 |
| rPCR | .004 | .002 | 188 | .000 | 5.64 | 5.22 | 41 | -74 | 47 |
| rSCR | .000 | .000 | 312 | .000 | 5.30 | 4.94 | 6 | -6 | 71 |
| lSCR | .000 | .000 | 268 | .000 | 5.18 | 4.85 | -5 | -14 | 74 |
| lSCR | .000 | .000 | 304 | .000 | 5.07 | 4.76 | -3 | 10 | 64 |
| MCP | .022 | .008 | 144 | .000 | 4.87 | 4.59 | -25 | -32 | -37 |
| lSCR | .007 | .003 | 173 | .000 | 4.80 | 4.53 | -31 | -9 | 69 |
| lSCR | .029 | .009 | 138 | .000 | 4.64 | 4.39 | -16 | -16 | 78 |
| lSCR | .030 | .009 | 137 | .000 | 4.18 | 4.00 | -30 | 15 | 59 |

The table presents the clusters demonstrating significant group differences in R1 values at the cluster level. FDR-corr: false-discovery rate-corrected. FWE-corr: family-wise error-corrected. HCs: healthy controls. lSCR: left superior corona radiata. K_E_: cluster extent. MCP: middle cerebellar peduncle. PwPD: patients with Parkinson’s disease. R1: longitudinal relaxation rate. rPCR: right posterior corona radiata. rSCR: right superior corona radiata. Uncorr: uncorrected. Z_E:_ equivalent z-scores.


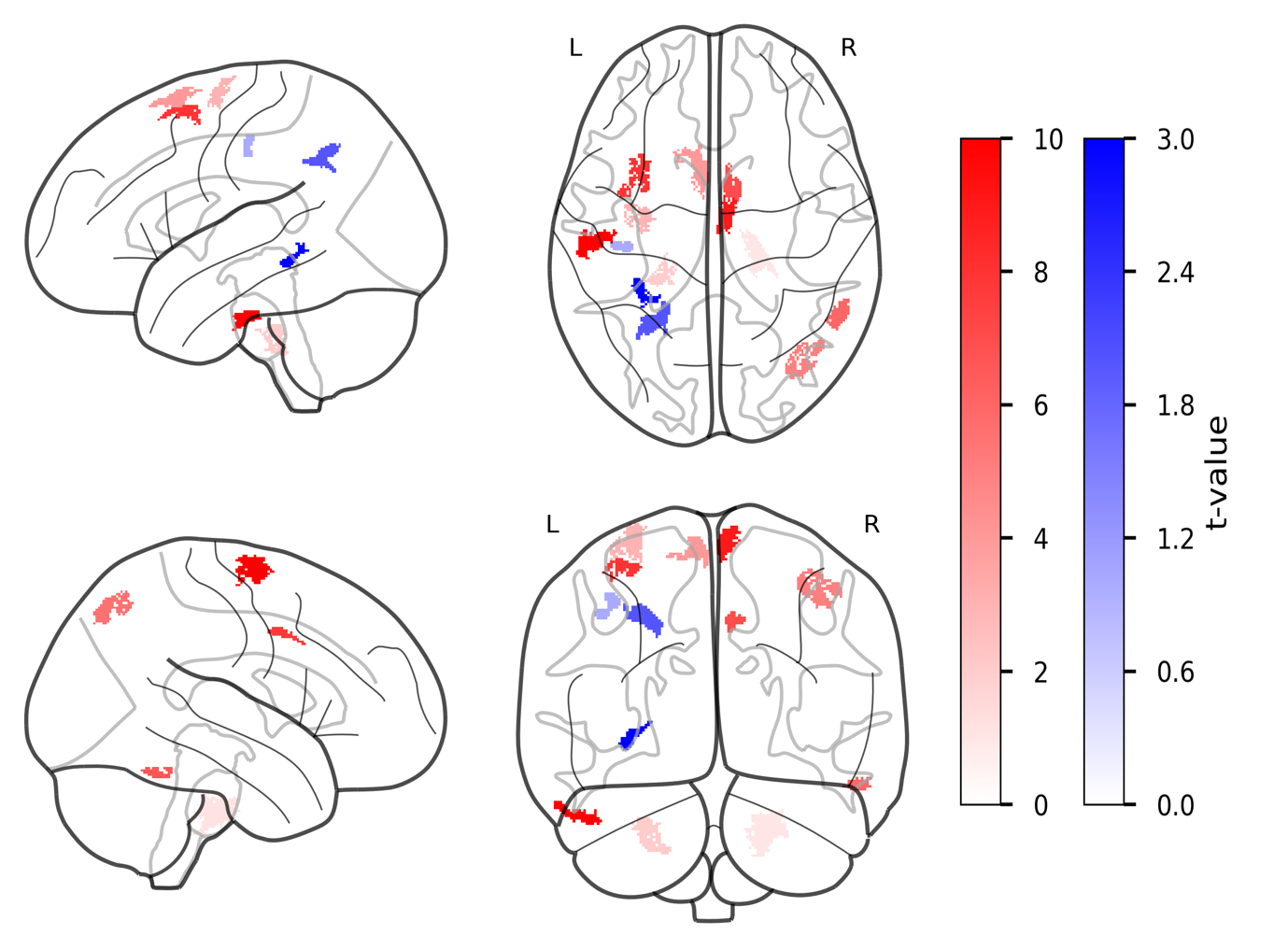


**Supplementary Figure 3. Group differences in R2* values between PwPD and HCs in white matter.** The statistical parametric maps from voxel-wise group comparison of R2* values are presented, displayed on a standard brain template in sagittal, axial, and coronal views. The statistical maps show t-statistics, where red clusters have higher R2* values in PwPD (PwPD > HCs), and blue clusters have higher R2* values in HCs (HCs > PwPD). The color bars reflect voxel-wise t-statistics, with higher absolute t-values indicating stronger group differences only. Cluster-defining threshold was set to T = 3.18 (p < .001, uncorrected) with a cluster extent threshold of k ≥ 20 voxels. FWE correction was applied at the cluster levels for PwPD > HCs contrast: FWEc = 154, df = [1, 94] and HCs > PwPD contrast: FWEc = 253, df = [1, 94]. df: degrees of freedom. FWE: family-wise error. HCs: healthy controls. k: number of voxels. L: left hemisphere. PwPD: patients with Parkinson’s disease. R: right hemisphere. R2*: effective transverse relaxation rate. T: t-statistic.

**Supplementary Table 2. Clusters with significant differences in R2* values between PwPD and HCs in white matter.**

|  | *cluster level* | | | | *peak level* | | *mm* | *mm* | *mm* |
| --- | --- | --- | --- | --- | --- | --- | --- | --- | --- |
|  | *P*_FWE-corr_ | *q*_FRD-corr_ | *K*_E_ | *P*_uncorr_ | *T* | (*Z*_E_) |  |  |  |
| **PwPD > HCs** | | | | | | | | | |
| MCP | .000 | .000 | 272 | .000 | 5.91 | 5.44 | 22 | -24 | -35 |
| rPCR | .006 | .002 | 168 | .000 | 5.56 | 5.16 | 41 | -74 | 47 |
| rSS | .021 | .006 | 140 | .000 | 5.49 | 5.10 | 56 | -49 | -22 |
| rSCR | .000 | .000 | 311 | .000 | 5.44 | 5.06 | 6 | -6 | 71 |
| lSCR | .000 | .000 | 275 | .000 | 5.22 | 4.88 | -5 | -14 | 74 |
| MCP | .035 | .008 | 129 | .000 | 5.10 | 4.78 | -25 | -32 | -37 |
| lSCR | .000 | .000 | 259 | .000 | 4.96 | 4.66 | -3 | 10 | 64 |
| rC | .025 | .007 | 136 | .000 | 4.96 | 4.66 | 7 | 6 | 41 |
| lSCR | .003 | .001 | 186 | .000 | 4.68 | 4.43 | -33 | -10 | 70 |
| lSS | .002 | .001 | 199 | .000 | 4.57 | 4.34 | -58 | -21 | -29 |
| lSCR | .006 | .002 | 167 | .000 | 4.28 | 4.08 | -31 | 10 | 60 |
| **HCs > PwPD** | | | | | | | | | |
| lSS | .019 | .003 | 142 | .000 | 4.26 | 4.06 | -31 | -38 | -5 |
| lSCR | .008 | .002 | 161 | .000 | 3.65 | 3.52 | -37 | -21 | 48 |
| lSCR | .000 | .000 | 416 | .000 | 3.59 | 3.47 | -29 | -54 | 42 |

The table presents the clusters demonstrating significant group differences in R2* values at the cluster level. FDR-corr: false-discovery rate-corrected. FWE-corr: family-wise error-corrected. HCs: healthy controls. lSCR: left superior corona radiata. lSS: left sagittal stratum. K_E_: cluster extent. MCP: middle cerebellar peduncle. PwPD: patients with Parkinson’s disease R2*: effective transversal relaxation rate. rC: right cingulum. rPCR: right posterior corona radiata. rSPC: right superior corona radiata. rSS: right sagittal stratum. Uncorr: uncorrected. Z_E:_ equivalent z-scores.


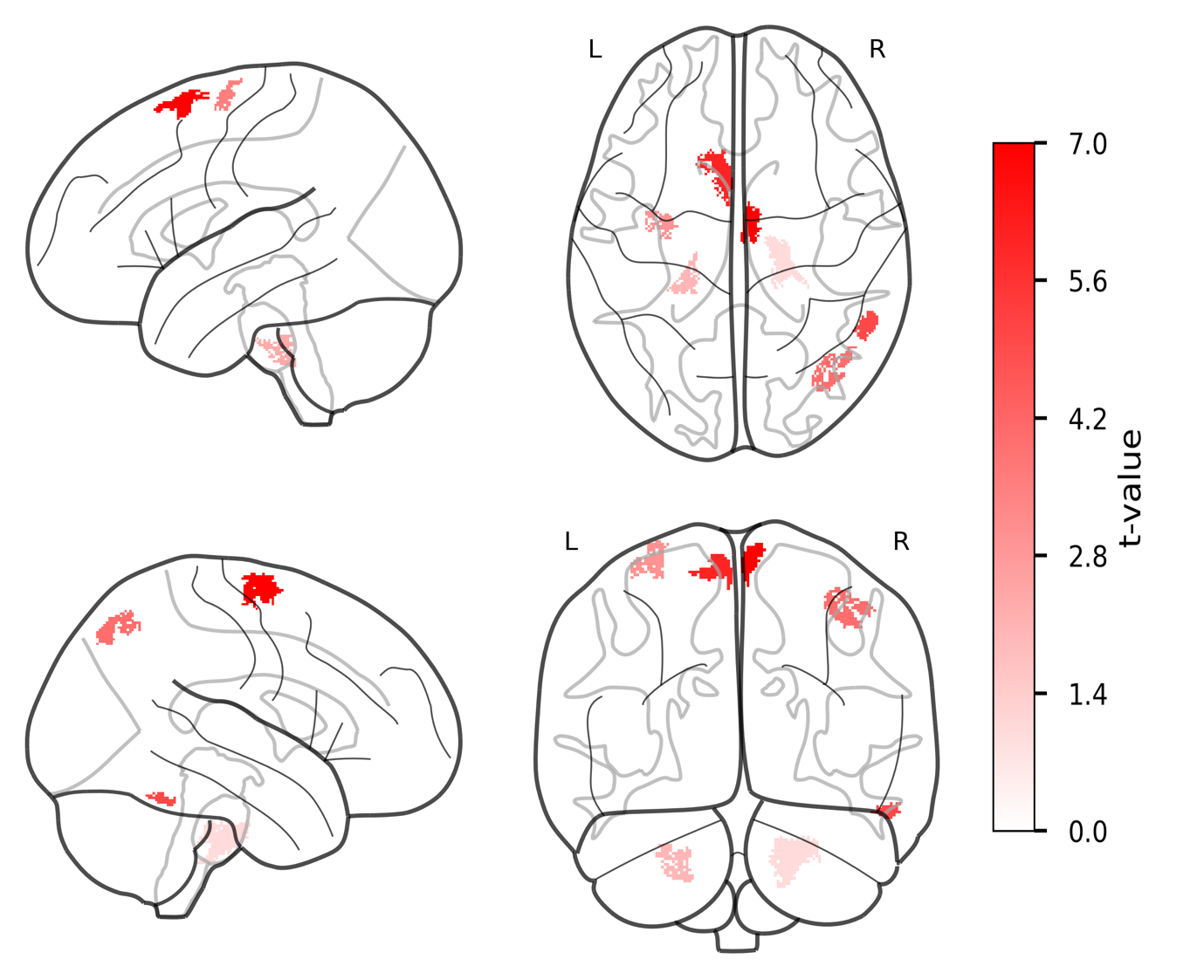


**Supplementary Figure 4. Group differences in proton density values between PwPD and HCs in white matter.**

The statistical parametric maps from voxel-wise group comparison of proton density values are presented, displayed on a standard brain template in sagittal, axial, and coronal views. The statistical maps show t-statistics, where red clusters have higher proton density values in PwPD (PwPD > HCs), and blue clusters have higher proton density values in HCs (HCs > PwPD). The color bars reflect voxel-wise t-statistics, with higher absolute t-values indicating stronger group differences only. Cluster-defining threshold was set to T = 3.18 (p < .001, uncorrected) with a cluster extent threshold of k ≥ 20 voxels. FWE correction was applied at the cluster levels for: FWEc = 122, df = [1, 94]. df: degrees of freedom. FWE: family-wise error. HCs: healthy controls. k: number of voxels. L: left hemisphere. PwPD: patients with Parkinson’s disease. R: right hemisphere. T: t-statistic.

**Supplementary Table 3. Clusters with significant differences in proton density values between PwPD and HCs in white matter.**

|  | *cluster level* | | | | *peak level* | | *mm* | *mm* | *mm* |
| --- | --- | --- | --- | --- | --- | --- | --- | --- | --- |
|  | *P*_FWE-corr_ | *q*_FRD-corr_ | *K*_E_ | *P*_uncorr_ | *T* | (*Z*_E_) |  |  |  |
| **PwPD > HCs** | | | | | | | | | |
| MCP | .000 | .000 | 351 | .000 | 5.81 | 5.36 | 22 | -24 | -35 |
| rPCR | .006 | .003 | 164 | .000 | 5.58 | 5.18 | 41 | -74 | 47 |
| rSS | .041 | .014 | 122 | .000 | 5.53 | 5.13 | 55 | -51 | -22 |
| rSCR | .000 | .000 | 348 | .000 | 5.44 | 5.06 | 6 | -6 | 71 |
| lSCR | .000 | .000 | 437 | .000 | 5.10 | 4.78 | -7 | -14 | 75 |
| lSCR | .000 | .000 | 312 | .000 | 5.04 | 4.73 | -8 | 15 | 67 |
| MCP | .022 | .008 | 135 | .000 | 4.83 | 4.56 | -25 | -32 | -37 |
| lSCR | .009 | .004 | 154 | .000 | 4.47 | 4.24 | -28 | -7 | 70 |

The table presents the clusters demonstrating significant group differences in proton density values at the cluster level. FDR-corr: false-discovery rate-corrected. FWE-corr: family-wise error-corrected. HCs: healthy controls. lSCR: left superior corona radiata. K_E_: cluster extent. MCP: middle cerebellar peduncle. PwPD: patients with Parkinson’s disease. rPCR: right posterior corona radiata. rSPC: right superior corona radiata. rSS: right sagittal stratum. Uncorr: uncorrected. Z_E:_ equivalent z-scores.


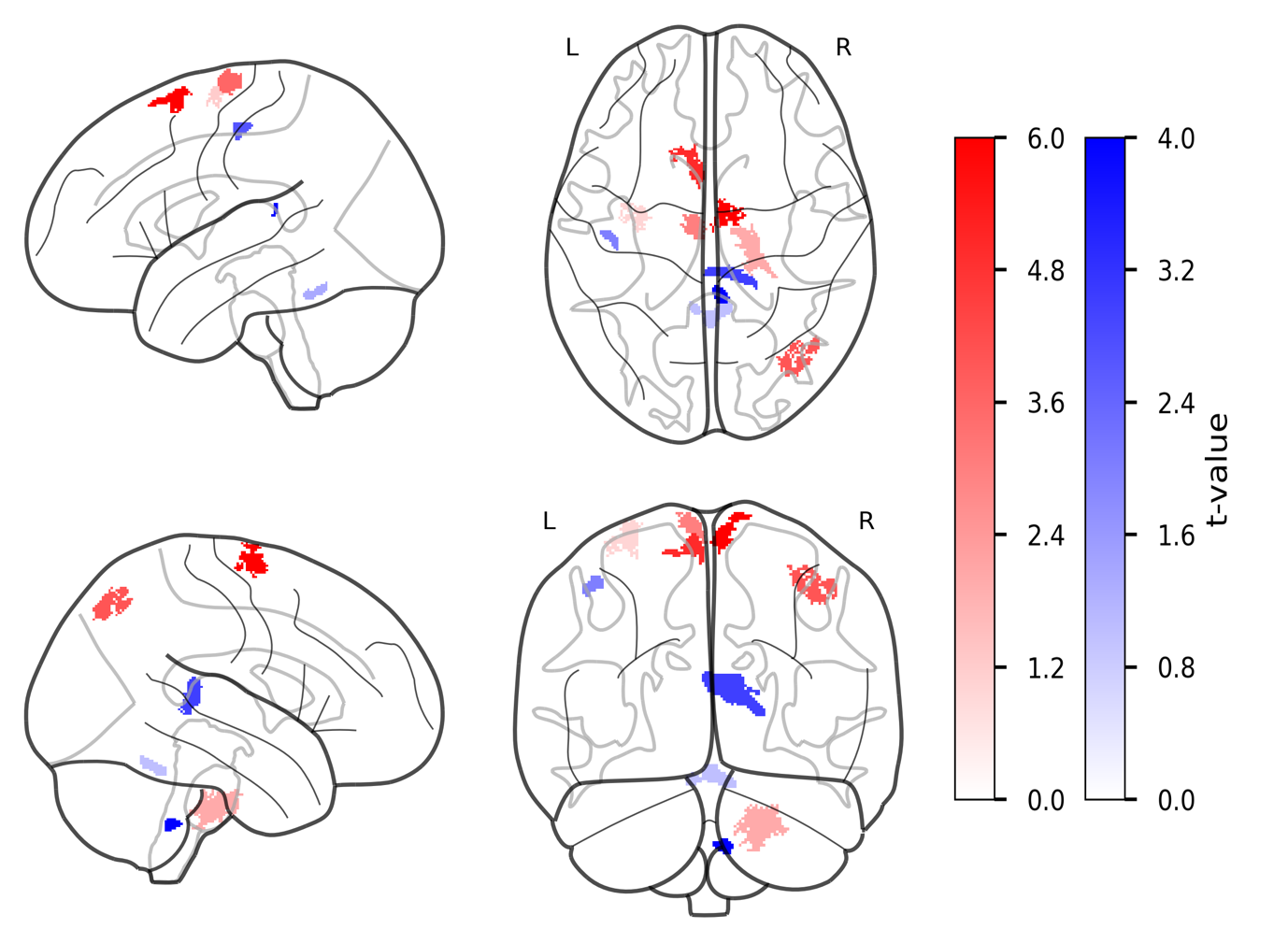


**Supplementary Figure 5. Group differences in MTsat values between PwPD and HCs in white matter.** The statistical parametric maps from voxel-wise group comparison of MTsat values are presented, displayed on a standard brain template in sagittal, axial, and coronal views. The statistical maps show t-statistics, where red clusters have higher MTsat values in PwPD (PwPD > HCs), and blue clusters have higher MTsat values in HCs (HCs > PwPD). The color bars reflect voxel-wise t-statistics, with higher absolute t-values indicating stronger group differences only. Cluster-defining threshold was set to T = 3.18 (p < .001, uncorrected) with a cluster extent threshold of k ≥ 20 voxels. FWE correction was applied at the cluster levels for PwPD > HCs contrast: FWEc = 167, df = [1, 94] and HCs > PwPD contrast: FWEc = 138, df = [1, 94]. df: degrees of freedom. FWE: family-wise error. HCs: healthy controls. k: number of voxels. L: left hemisphere. MTsat: magnetic transfer saturation. PwPD: patients with Parkinson’s disease. R: right hemisphere. T: t-statistic.

**Supplementary Table 4. Clusters with significant differences in MTsat values between PwPD and HCs in white matter.**

|  | *cluster level* | | | | *peak level* | | *mm* | *mm* | *mm* |
| --- | --- | --- | --- | --- | --- | --- | --- | --- | --- |
|  | *P*_FWE-corr_ | *q*_FRD-corr_ | *K*_E_ | *P*_uncorr_ | *T* | (*Z*_E_) |  |  |  |
| **PwPD > HCs** | | | | | | | | | |
| MCP | .000 | .000 | 340 | .000 | 5.74 | 5.31 | 22 | -24 | -35 |
| rPCR | .005 | .002 | 187 | .000 | 5.59 | 5.19 | 41 | -74 | 47 |
| rSCR | .000 | .000 | 273 | .000 | 5.19 | 4.86 | 55 | -51 | -22 |
| lSCR | .001 | .001 | 230 | .000 | 5.06 | 4.75 | 6 | -6 | 71 |
| lSCR | .002 | .001 | 209 | .000 | 4.67 | 4.42 | -7 | -14 | 75 |
| lSCR | .010 | .004 | 167 | .000 | 4.62 | 4.38 | -8 | 15 | 67 |
| **HCs > PwPD** | | | | | | | | | |
| sCC | .000 | .000 | 525 | .000 | 4.24 | 4.05 | -20 | -42 | 12 |
| rICP | .000 | .000 | 391 | .000 | 3.82 | 3.68 | 1 | -55 | -18 |
| lSLF | .034 | .006 | 118 | .000 | 3.73 | 3.59 | -43 | -17 | 53 |
| rPCR | .020 | .005 | 151 | .000 | 3.68 | 3.55 | 6 | -43 | -46 |

The table presents the clusters demonstrating significant group differences in MTsat values at the cluster level. FDR-corr: false-discovery rate-corrected. FWE-corr: family-wise error-corrected. HCs: healthy controls. lSCR: left superior corona radiata. lSLF: left superior longitudinal fasciculus. K_E_: cluster extent. MCP: middle cerebellar peduncle. PwPD: patients with Parkinson’s disease. rICP: right inferior cerebellar peduncle. rPCR: right posterior corona radiata. rSPC: right superior corona radiata. sCC: splenium of corpus callosum. Uncorr: uncorrected. Z_E:_ equivalent z-scores.

**
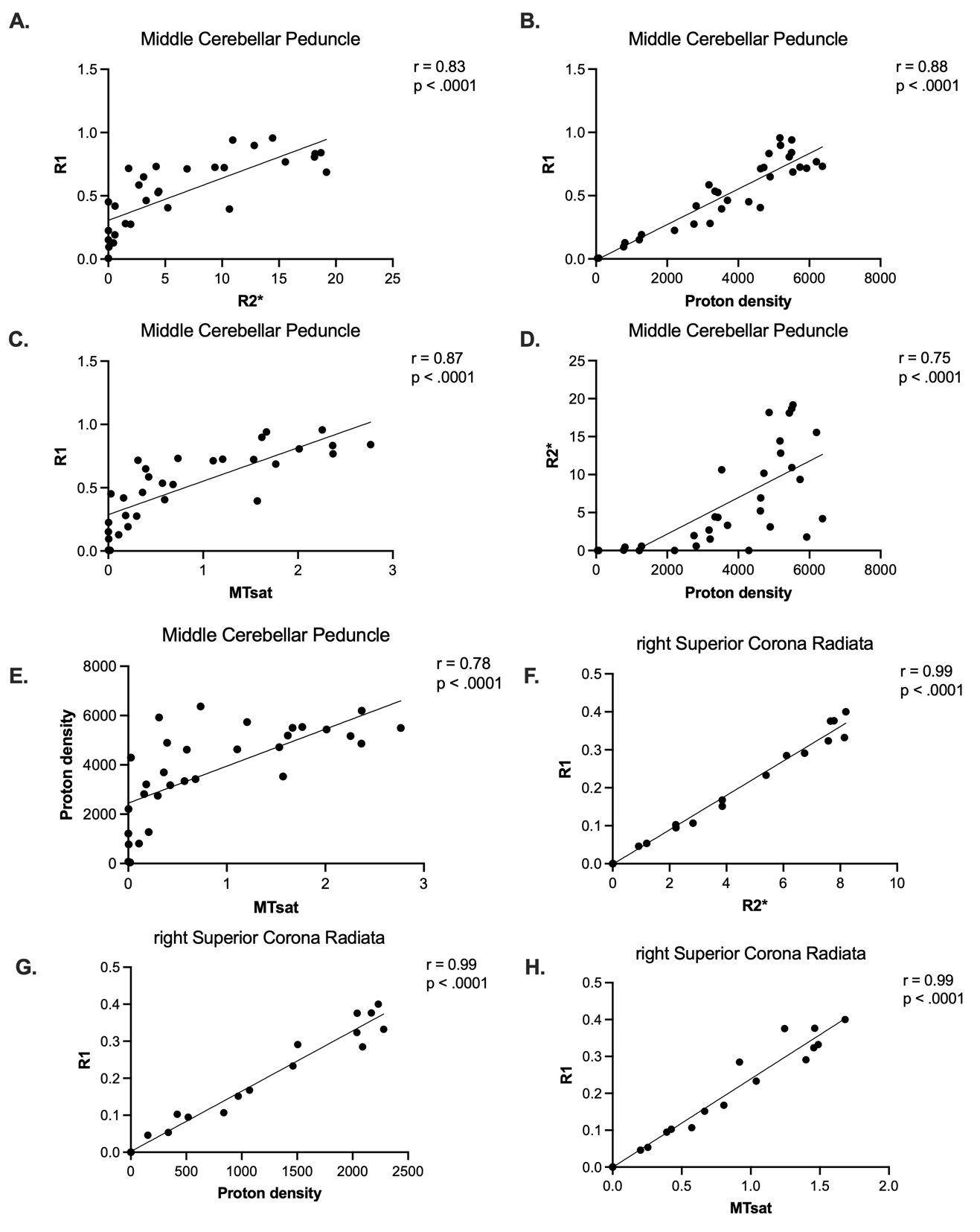
**

**Supplementary Figure 6. Scatter plots of cross-modal correlation between mean MPM parameter values in clusters with significant group differences in white matter.** The figure presents multiple panels with scatter plots and key statistical data assessing the correlation between mean MPM-derived parameters from overlapping clusters with significant differences between PwPD and HCs in the middle cerebellar peduncle (A-E) and right superior corona radiata (F-H). Only the clusters that passed the Bonferroni correction (p < .00625) were included in the presented analysis. HCs: healthy controls. MPM: multiparametric mapping. MTsat: magnetic transfer saturation. PwPD: patients with Parkinson’s disease. R1: longitudinal relaxation rate. R2*: effective transverse relaxation rate.

**
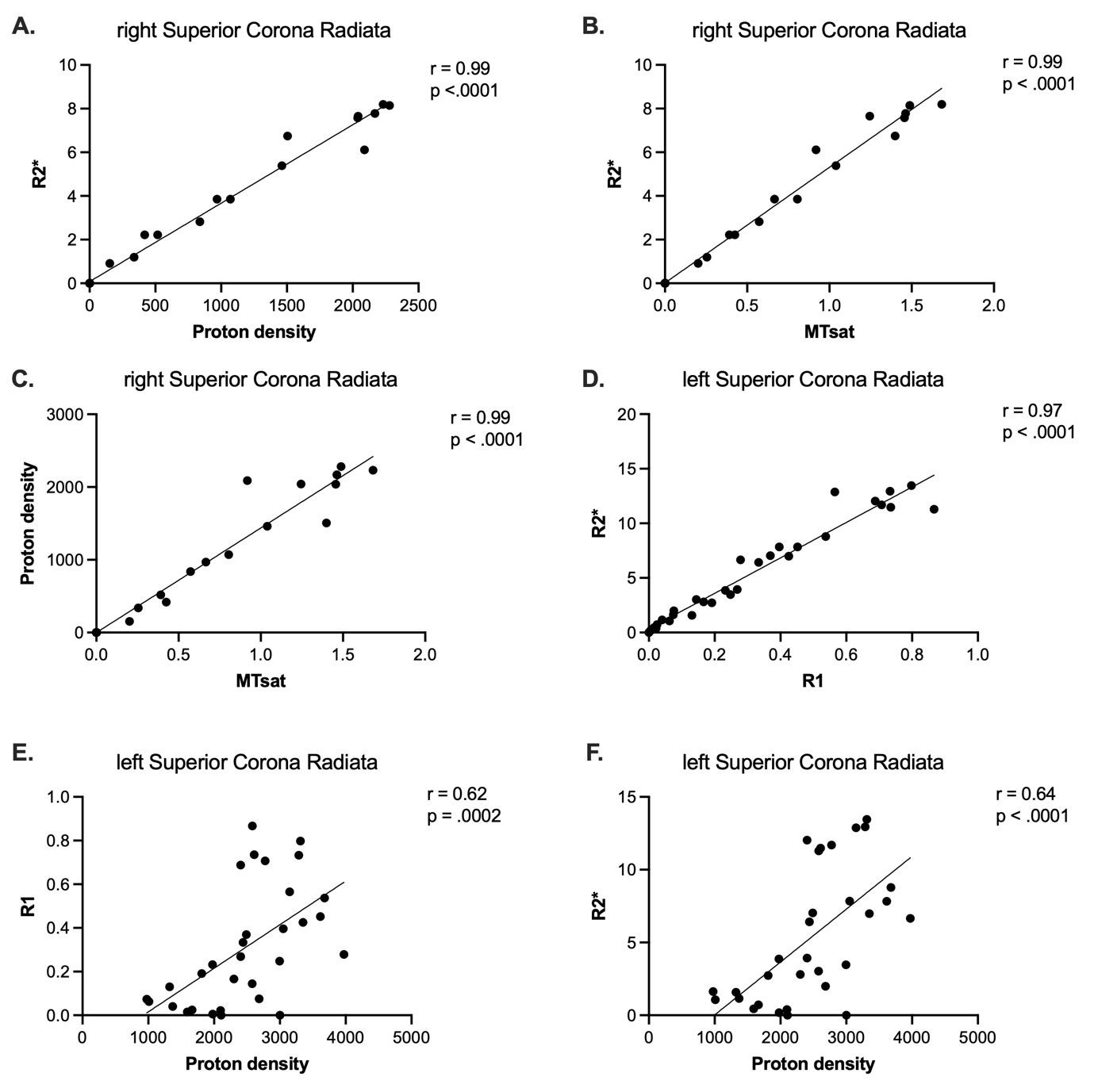
**

**Supplementary Figure 7. Scatter plots of cross-modal correlation between mean MPM parameter values in clusters with significant group differences in white matter.** The figure presents multiple panels with scatter plots and key statistical data assessing the correlation between mean MPM-derived parameters from overlapping clusters with significant differences between PwPD and HCs in bilateral superior corona radiata (right: A-D and left: E and F). Only the clusters that passed the Bonferroni correction (p < .00625) were included in the presented analysis. HCs: healthy controls. MPM: multiparametric mapping. MTsat: magnetic transfer saturation. PwPD: patients with Parkinson’s disease. R1: longitudinal relaxation rate. R2*: effective transverse relaxation rate.

**Supplementary Figure 8. Venn-encoded overlap of identified group differences across MPM modalities in white matter.** The figure illustrates the clusters with identified significant group-based differences in biophysical properties informed by MPM modalities, including R1, R2*, proton density, and MTsat. Identified clusters are color-coded depending on the number of MPM modalities with identified differences. HCs: healthy controls. L: left hemisphere. MPM: multiparametric mapping. MTsat: magnetic transfer saturation. PwPD: patients with Parkinson’s disease. R: right hemisphere. R1: longitudinal relaxation rate. R2*: effective transverse relaxation rate.
